# A Polyphasic Approach for Phenotypic and Genetic Characterization of the Fastidious Aquatic Pathogen *Francisella noatunensis* subsp. *orientalis*

**DOI:** 10.3389/fmicb.2017.02324

**Published:** 2017-12-12

**Authors:** José G. Ramírez-Paredes, Kim D. Thompson, Matthijs Metselaar, Khalid Shahin, Esteban Soto, Randolph H. Richards, David J. Penman, Duncan J. Colquhoun, Alexandra Adams

**Affiliations:** ^1^Faculty of Natural Sciences, Institute of Aquaculture, University of Stirling, Stirling, United Kingdom; ^2^Aquaculture Research Group, Moredun Research Institute, Edinburgh, United Kingdom; ^3^The Fish Vet Group, Inverness, United Kingdom; ^4^Department of Medicine and Epidemiology, School of Veterinary Medicine, University of California, Davis, Davis, CA, United States; ^5^Section for Bacteriology, Norwegian Veterinary Institute, Oslo, Norway

**Keywords:** tilapia diseases, Francisellosis in tilapia, *Francisella noatunensis* subsp. *orientalis*, OMVs, *Fno* antimicrobial resistance, *Francisella* characterization

## Abstract

*Francisella noatunensis* subsp. *orientalis* (*Fno*) is the causative agent of piscine francisellosis, an emerging infectious disease in Asia and Latin America. In this study two outbreaks of francisellosis were diagnosed in the UK on the basis of histopathology, electron microscopy, PCR, bacterial isolation and fulfillment of Koch's postulates. Furthermore, a phenotypic fingerprint based on biochemical analyses, metabolic activity, chemotaxonomic composition, and antimicrobial assays was generated for the novel isolates, the *Fno* type strain Ehime-1 from Asia and other *Fno* from Latin America. The genetic relatedness between the novel *Fno* and other *Francisellaceae* species was investigated by sequencing and comparing the 16SrRNA gene, 8 housekeeping genes (individually and concatenated) and the 16SrRNA-ITS-23SrRNA sequence. The phenotypic profiling indicated a high degree of similarity among the *Fno* strains as all were able to metabolize dextrin, N-acetyl-D glucosamine, D-fructose, α-D-glucose, D-mannose, methyl pyruvate, acetic acid, α-keto butyric acid, L-alaninamide, L-alanine, L-alanylglycine, L-asparagine, L-glutamic acid, L-proline, L-serine, L-threonine, inosine, uridine, glycerol, D L-α-glycerol phosphate, glucose-1-phosphate, and glucose-6-phosphate. The chemotaxonomic analyses indicated that 24:1 (20.3%), 18:1n-9 (16.9%), 24:0 (13.1%) 14:0 (10.9%), 22:0 (7.8%), 16:0 (7.6%), and 18:0 (5.5%) were the predominant structural fatty acids in *Fno*. The antimicrobial assays showed little variation between the isolates and high susceptibility to enrofloxacin, gentamicin, neomycin, streptomycin, amikacin, ciprofloxacin, gatifloxacin, nitrofurantoin, tobramycin, kanamycin, tetracycline, oxytetracycline, florfenicol, oxolinic acid, and streptomycin in all the *Fno* analyzed. In all the phylogenetic trees the *Fno* strains clustered together in independent branches confirming a high degree of homogeneity. Interestingly in five of the 11 trees i.e., *mutS, putA, rpoB*, 16SrRNA-ITS-23SrRNA, and concatenated sequence the two *Francisella noatunensis* ssp. diverged more from each other than from the closely related *Francisella philomiragia* (*Fp*). The phenotypic and genetic characterization confirmed the *Fno* isolates represent a solid phylo-phenetic taxon that in the current context of the genus seems to be misplaced within the species *Fn*. We propose the use of the present polyphasic approach in future studies to characterize strains of *Fnn* and *Fp* and verify their current taxonomic rank of *Fno* and other aquatic *Francisella* spp.

## Introduction

*Francisella noatunensis* (*Fn*) (family *Francisellaceae*) is a Gram negative, non-motile, non-sporulating, aerobic, facultative intracellular coccobacillus that causes “piscine francisellosis,” a disease that affects farmed and wild marine and fresh water fish species worldwide (Colquhoun et al., 2014). The closest related species to *Fn* is *Francisella philomiragia* (*Fp*) a non-fastidious aquatic bacteria frequently isolated around brackish or sea water environments that can act as an opportunistic pathogen and naturally infect immunosuppressed mammals such as muskrats, dogs, and humans (Hollis et al., 1989; Wenger et al., 1989; Ender and Dolan, 1997; Whipp et al., 2003; Friis-Møller et al., 2004; Mailman and Schmidt, 2005; Berrada and Telford, 2010; Cora et al., 2010; Whitehouse et al., 2012; Kreitmann et al., 2015; Relich et al., 2015).

At present *Fn* is divided into two subspecies: *noatunensis* (*Fnn*) and *orientalis* (*Fno*) (Ottem et al., 2009) of which the former is responsible for disease in cold water fish, principally farmed and wild Atlantic Cod (*Gadus morhua* L.) and farmed Atlantic salmon (*Salmo salar* L.) in sea and fresh water respectively, whereas the latter causes disease in a wide range of warm, marine, and fresh water fish (Birkbeck et al., 2011; Colquhoun and Duodu, 2011).

*Francisella noatunensis* is a highly fastidious pathogen that grows slowly and requires complex artificial selective media for its isolation. The recovery of *Francisella* from fish has, therefore, been historically challenging, and several cases of unspeciated *Francisella* spp. and *Francisella*-like bacteria (FLB) have been reported based on non-culture molecular studies (Ostland et al., 2006; Hsieh et al., 2007; Jeffery et al., 2010). A recent study by Assis et al. (2017) has documented the limitations and low sensitivity of the culture method as a diagnostic tool and up until now the diagnosis of piscine francisellosis often relies in the molecular identification of the bacteria in the fish tissues.

For these reasons very limited information is available on the phenotypic characteristics of *Fn* and these circumstances have also complicated the historic nomenclature and taxonomy of these bacteria. Initially in July 2007, a comparative characterization of a single *Francisella* sp. strain recovered from farmed Norwegian Atlantic cod (*G. morhua*) was made with the type strain of *F. philomiragia*, in that study the cod isolate was classified as a novel species named *F. piscicida* (Ottem et al. (2007b).

A few months later in September 2007, after the name *F. piscicida* had been published but not yet validated, Mikalsen et al. (2007) compared seven cod isolates against four strains of *Fp* (including the type strain) and validly published a new nomenclature of the bacterium i.e., *F. philomiragia* subsp. *noatunensis* (Euzeby, 2007). In January 2008, *F. piscicida* was published as a valid species (Euzeby, 2008) and because of the similarity of its 16S rRNA gene the bacterium was considered to be a heterotypic synonym of *F. philomiragia* subsp. *noatunensis* thus, according to the rule of priority, the epithet “*noatunensis*” remained over “*piscicida*.”

Later the same year, the current taxonomical status of fish *Francisella* was revised by Ottem et al. (2009). In that study, the type strains of *F. piscicida* and its heterotypic synonym *F. philomiragia noatunensis* were compared against each other and five isolates of *Fp* including the type strain. Additionally, one strain from a diseased fish farmed in warm water environment in Japan i.e., Ehime-1 and DNA from one strain recovered in Indonesia (Ind04/Toba04) were included. As a result of those comparisons *F. piscicida* was shown to be the heterotypic synonym of *F. philomiragia* subsp. *noatunensis* and both were elevated to the rank of species as *F. noatunensis*, while the strain Ehime-1 was described as the type strain of the new subspecies *F. noatunensis orientalis* on the basis of very limited phenotypic traits. These results appeared to have elucidated the new nomenclature for the various isolates, but were not considered as valid until published in the “list of new names and new combinations previously effectively, but not validly, published” in 2009 (Euzeby, 2009a,b).

In September 2009, an “ahead of print electronic publication” of the International Journal of Systematic and Evolutionary Microbiology (IJSEB) appeared online. In this study a Chilean strain named “PQ1106,” DNA from the Japanese strain Ehime-1 and a strain from tilapia farmed in Costa Rica were analyzed. The result of this study also elevated the rank *F. philomiragia noatunensis* to the species level as *F. noatunensis* and additionally described the Costa Rican and Japanese strains as a new species for which the authors gave the name *F. asiatica* (Mikalsen and Colquhoun, 2009). Because *F. noatunensis* had already been published as a valid name, this electronic version of the paper was later withdrawn by the IJSEB and the name *F. asiatica* was never considered as valid or even effectively published. Since the validation of *F. noatunensis*, no further studies have been performed to investigate the phenotypic characteristics of these bacteria.

In Europe outbreaks of francisellosis caused by *Fnn*, have been reported in Atlantic cod and Atlantic salmon in Norway, Sweden, the United Kingdom (UK), Denmark, and Ireland (Ottem et al., 2007b, 2008; Zerihun et al., 2011; Ruane et al., 2013), while *Fno* has been diagnosed using molecular methods in Nile tilapia (*Oreochromis niloticus* L.) in the UK (Jeffery et al., 2010) and isolated from ornamental Malawi cichlids in Austria (Lewisch et al., 2014). Recently, the isolation of novel *Fn* strains, particularly of *Fno*, has been reported in Asia and Latin America (Leal et al., 2014; Lin et al., 2015; Nguyen et al., 2016; Ortega et al., 2016), however these studies also lack in-depth methodology for the integrated characterization of the bacteria, as their identifications were based on few primary phenotypic and genetic characteristics.

The aims of this study were to diagnose and characterize outbreaks of granulomatous disease in Nile tilapia (*O. niloticus*) farmed in the UK, isolate novel *Fno* strains and generate a comprehensive methodology for their characterization and investigate the genetic relatedness between the novel *Fno* and other *Francisella* spp.

## Materials and methods

### Clinical samples and associated diagnostics

#### Sampling of diseased tilapia

During 2011 and early 2012, chronic disease episodes characterized by largely non-specific clinical signs and mortalities of up to 60% were experienced in farmed red and wild type Nile tilapia fingerlings at two farms in Lincolnshire UK. In July 2012, five fish from each farm were randomly collected with a net, including apparently healthy and fish showing clinical sings from different sections of the farm and different sizes. The fish were euthanized with a lethal overdose of Tricaine methanesulfonate 1,000 mg/g (TPQ) (Pharmaq, Hampshire, UK), and necropsied. Samples of gills, heart, kidney, liver, and spleen were aseptically collected and fixed in 10% (v/v) neutral buffered formalin, 2.5% (v/v) glutaraldehyde in 100 mM sodium cacodylate buffer (pH 7.2) and 96% ethanol for histology, electron microscopy, and molecular diagnosis, respectively. The samples were sent for diagnosis to the Aquatic Vaccine Unit at Institute of Aquaculture (IoA), University of Stirling (UoS).

#### Histopathological, transmission (TEM), and scanning electron microscopy (SEM) analyses

Formalin fixed tissues were processed using standard histological methods, stained with haematoxylin and eosin (H&E) and examined at 40x and 200x magnification on a Olympus BX51 light microscope (Olympus, Tokyo, Japan) equipped with a AxioCam MRc digital camera (Carl Zeiss, Göttingen, Germany). Glutaraldehyde fixed spleen and head kidney tissues were processed using standard methods for TEM and SEM. The sections were observed under an FEI Tecnai Spirit G2 Bio Twin TEM and a Jeol JSM6460LV SEM.

#### Molecular diagnosis using a genus specific PCR

A *Francisella* genus specific PCR (Forsman et al., 1994) was performed using total genomic DNA (gDNA) from the fish sampled in July 2012 as a template. Previous studies have validated the use of this PCR as an inexpensive and practical tool to identify *Francisella* spp. in fresh and archived fish tissues (Hsieh et al., 2006; Soto et al., 2009).

The gDNA was extracted from the ethanol fixed spleens using a Nucleo Spin Tissue® kit (Macherey & Nagel, Düren, Germany) according to the manufacturer's instructions. A negative control was included using gDNA from tilapia reared at the Tropical Aquarium (TA) IoA-UoS. The TA was confirmed to be a source free of francisellosis after sampling 25 fish for bacteriological (culture in plates) and molecular (PCR) diagnosis. The PCR was performed using Illustra PuReTaq Ready-To-Go Beads™ (200 μM each dNTP in 10 mM Tris-HCl, pH 9.0, 50 mM KCl, and 1.5 mM MgCl2) (GE Healthcare, Chalfont St. Giles, UK) reconstituted to a final volume of 25 μl with: 5 μl of DNA template (~80 ng/μl), 2.5 μl of each primer (F11 5′-TAC CAG TTG GAA ACG ACT GT-3′ and F5 5′-CCT TTT TGA GTT TCG CTC C-3′) at a concentration of 10 pmol/ml (10 μM) and 15 μl of ultrapure water. Cycling conditions consisted of an initial denaturation step of 2 min at 93°C, followed by 35 cycles of: 1 min at 94°C, 1 min at 65°C, and 1 min at 72°C, and a final extension step of 5 min at 72°C in a Biometra TGradient Thermocycler (Biometra, Göttingen, Germany). Amplification products were visualized on ethidium bromide stained 1% agarose gel after electrophoresis for 35 min at 90 V.

### Bacterial isolation

#### Primary isolation and purification

During a follow up visit to the farms in November 2012, 10 red Nile tilapia from Farm 1 and 8 red and 2 wild type from Farm 2, were randomly sampled including apparently healthy fish as well as individuals showing clinical sings from different sections of the farms. The fish spleens were aseptically collected and half of them homogenized in 1 ml of 1x sterile phosphate buffered saline (0.02 M phosphate, 0.15 M Na Cl, and pH adjusted at 7.2) (PBS) using a Cordless Motor Pellet Pestle (Sigma-Aldrich, Dorset, UK). The other half of the spleens and all the kidneys were fixed in 96% ethanol and screened with the genus specific PCR previously described. To achieve primary isolation 5 different media were evaluated. Thus, ~20 μl of the spleen homogenates were streaked onto: cystine heart agar complemented in a 50% solution (v/v) with 2% bovine hemoglobin (**CHAH**; BD, Oxford, UK), modified Martin Lewis agar (**MMLA**; BD, Oxford, UK), modified Thayer-Martin Agar, (**MTMA**; BD, Maryland, USA), tryptone soya agar (**TSA**; Oxoid Ltd., Hampshire, UK), and cystine heart agar supplemented with 5% (v/v) tilapia blood (**CHTB**). In addition CHAH and CHTB were prepared with and without polymyxin B sulfate salt 100 units/ml (Sigma-Aldrich, Dorset, UK) and Ampicillin Ready Made Solution 50 μg/ml (Sigma-Aldrich, Dorset, UK).

All inoculated plates were incubated at 28°C for 10 days. For purification, single smooth, convex, circular colonies with a greenish-grayish color were subcultured twice on CHAH at the same temperature as for isolation. Gram staining, catalase and oxidase production, oxidation/fermentation of glucose (O/F test), and motility tests were carried out using standard methods.

Suspected *Francisella* like colonies (smooth, convex, with a greenish-grayish color) were grown in Modified Mueller-Hinton II cation adjusted broth supplemented with 2% IsoVitaleX (BD, Oxford, UK) and 0.1% D-(+)-glucose ACS reagent (Sigma-Aldrich, Dorset, UK) (**MMHB**). The broth cultures were grown overnight at 28°C in a shaker incubator at 175 rpm and stored at −80°C with 20% sterilized glycerol (BDH Prolabo-VWR International Eurolab, Leuven, Belgium). To confirm the identity of the colonies, their genomic DNA was obtained using the boiling technique outlined by Seward et al. (1997) with modifications. Briefly, five bacterial colonies were re-suspended in a 1.5 ml Eppendorf tube containing 100 μl of Tris-EDTA (TE) buffer and heated for 10 min at 99°C. The suspensions were then cooled on ice for 5 min and centrifuged at 15,800 g in a benchtop IEC Microlite Centrifuge (Thermo Electron Corporation, Massachusetts, USA) for 1 min. The supernatant containing the crude gDNA was then transferred into a fresh 0.5 ml tube and used as template in the genus specific PCR previously described.

### Phenotypic characterization

#### Bacterial isolates and growth conditions

Of the isolates recovered from the diseased fish, two isolates i.e., STIR-MATT-F1-f6 (from Farm 1) and STIR-GUS-F2f7 (from Farm 2), confirmed as *Francisella* spp. by PCR, were selected as representative of the outbreaks for further phenotypic analyses. The *Fno* type strain Ehime-1 (DSM 21254), *Fno* PQ1104 (from different geographical origin) and *Escherichia coli* ATCC 25922 were included as controls. All *Fno* isolates were grown on CHAH and MMHB, whereas *E. coli* was cultured on TSA and in tryptone soya broth (TSB; Oxoid Ltd., Hampshire, UK). *Fno* was cultured on solid media for 96 h, and *E. coli* for 24 h. Broth cultures were incubated in 15 ml aliquots for 18–21 h (i.e., mid log phase of the growth curve) in a shaker incubator at 150 rpm. All isolates were grown at 28°C. The *Fno* isolates used and recovered in this study are presented in Table 1.

**Table 1:** *Francisella noatunensis orientalis* isolates used and recovered in this study.

**Isolate**	**Location**	**Year**	**Diseased fish**
Ehime-1	Japan	2001	Three line grunt or Isaki fish
			(*Parapristipoma trilineatum*)
PQ1104	Costa Rica	2006	Tilapia (*Oreochromis* sp.)
STIR-AVU-F1f3	UK farm 1 fish 3	2012^*^	Red Nile tilapia (*O. niloticus*)
STIR-AVU-F1f4	UK farm 1 fish 4	2012^*^	Red Nile tilapia (*O. niloticus*)
STIR-AVU-F1f5	UK farm 1 fish 5	2012^*^	Red Nile tilapia (*O. niloticus*)
STIR-MATT-F1f6	UK farm 1 fish 6	2012^*^	Red Nile tilapia (*O. niloticus*)
STIR-AVU-F1f7	UK farm 1 fish 7	2012^*^	Red Nile tilapia (*O. niloticus*)
STIR-AVU-F1f9	UK farm 1 fish 9	2012^*^	Red Nile tilapia (*O. niloticus*)
STIR-AVU-F1f10	UK farm 1 fish 10	2012^*^	Red Nile tilapia (*O. niloticus*)
STIR-GUS-F2f7	UK farm 2 fish 7	2012^*^	Red Nile tilapia (*O. niloticus*)
STIR-AVU-F2f9	UK farm 2 fish 9	2012^*^	Red Nile tilapia (*O. niloticus*)
STIR-AVU-F2f10	UK farm 2 fish 10	2012^*^	Red Nile tilapia (*O. niloticus*)

* *From present study*.

#### Optimal growth *in vitro* and growth curves

The optimal *in vitro* growth temperature of each *Fno* isolate was investigated on agar by plating in triplicate six 20 μl drops containing the same bacterial concentration (dilution 10^−6^) with an optical density of 0.4 at a wavelength of 600 nm (OD_600_ 0.4) and incubating them at 5, 15, 18, 21, 22, 24, 26, 28, 29, 30, 32, 33, and 37°C for 10 days.

Growth curves were established for STIR-GUS-F2f7 and Ehime-1 by inoculating and incubating triplicate flasks containing 99 ml of MMHB with 1 ml of starting culture (OD_600_ 1.0) for 72 h at 28°C, on an orbital shaker incubator at 150 rpm. To monitor growth, a 1 ml sample was taken every 3 h and its optical density recorded. The bacterial growth curve was produced by plotting the absorbance of the culture at 600 nm against time (h).

#### Carbohydrate fermentation and enzymatic activity

Enzymatic activity and fermentation of carbohydrates was assessed for each isolate in triplicate using API 20E and API ZYM kits (BioMerieux, Marcy l'Etoile, France). The kits were used according to the manufacturer's instructions with the following modifications: bacteria were precultured in CHAH at 28°C, the API ZYM kit-strips were visually read at 4, 8, and 24 h post inoculation (hpi) and the API20E strips after 24 hpi.

#### Carbon metabolism (metabolic fingerprint)

To assess the metabolic activity (carbon utilization) of *Fno*, three Biolog-GN2 microplates (Biolog Inc., California, USA) were used per each strain. The plates were set up and analyzed according to the following protocol: bacteria grown on CHAH, as previously described. For each microplate, three 15 ml aliquots of MMHB were prepared in 50 ml centrifuge tubes. After incubation at 28°C for 18–21 h, the cultures were centrifuged in a Sigma 4K15C refrigerated benchtop centrifuge (Sigma Laborzentrifugen GmbH, Osterode am Harz, Germany) at 4°C for 15 min at 2,602 × g. The bacterial pellets were then washed by re-suspending them in 20 ml of sterile PBS (a sterile disposable 5 μl loop was required to break resuspend the pellets) and vortexing for 5 min. The tubes were centrifuged as described before and the PBS discarded. Each pellet was then suspended in 3 ml of GN inoculating fluid (Biolog Inc., California, USA) in a 50 ml centrifuge tube. In order to optimize the protocol i.e., identify the optimal inoculation density, a gradient of bacterial concentrations was prepared for the type strain Ehime-1 and STIR-GUS-F2f7 using the GN inoculating fluid. The OD_600_ tested were 0.36, 0.46, 0.56, 0.66, 0.76, 0.86, 0.96, 1.06, and 1.5.The plates were inoculated with 150 μl of the adjusted bacterial suspension per well, using a multichannel pipette and a sterile reservoir (Biolog Inc., California, USA). After inoculation, the Biolog GN2 microplates were incubated at 28°C for 24 h and the color change recorded every 3 h by visual inspection. Following preliminary results from the density gradient, the remaining *Fno* isolates were subsequently tested at an OD_600_ 0.85. The criteria chosen for the selection of this value was: the higher density at which the negative control remains as negative.

#### Cellular fatty acids methyl esters analyses

The cellular fatty acid methyl esters (FAME) composition of *Fno* was analyzed by gas chromatography (GC) according to the protocol established by Tocher and Harvie (1988). The two representative *Fno* isolates and the type strain Ehime-1 were grown in MMHB as previously described, using three 50 ml centrifuge tubes containing 20 ml of MMHB per isolate, and incubated for 43 h. After incubation, the absorbance of the cultures at 600 nm was determined and the bacterial suspension centrifuged at 4°C for 15 min at 2,602 × g. The resulting bacterial pellets were then washed by re-suspending them in 5 ml of sterile PBS, vortexing for 5 min and centrifuging at 4°C for 15 min at 2,602 × g.

The lipid content was extracted by suspending the pellets in 5 ml of ice cold chloroform/methanol (2:1 v/v) using a disposable glass Pasteur pipette and quantified gravimetrically. FAME were prepared by acid catalyzed trans-esterification at 50°C for 17 h. FAME were extracted (not purified) from the total lipid content and separated and quantified by GC using a Fisons GC-8160 (Thermo Scientific, Milan, Italy) equipped with a 30 m × 0.32 mm × 0.25 mm ZB-wax column (Phenomenex, Cheshire, UK) “on column” injection and flame ionization detection. Hydrogen was used as carrier gas with an initial oven thermal gradient from 50 to 150°C at 40°C per min to a final temperature of 230°C at 2°C per min. Individual FAME were identified by comparison to known standards i.e., Supelco™ 37-FAME mix (Sigma-Aldrich, Dorset, UK). Data were collected and processed using Chromcard version 1.19 (Thermoquest Italia SpA., Milan, Italy).

### Antibacterial susceptibility tests

#### Broth microdilution method

The minimal inhibitory concentration (MIC) of 39 different antimicrobial compounds was investigated using GN2F and AVIAN1F Sensititre® Plates (Trek Diagnostic System, West Sussex, UK). This procedure was performed in duplicate following the manufacturer's instructions and previously published protocols for *Fno* and *Francisella tularensis* (Baker et al., 1985; Brown et al., 2004; García del Blanco et al., 2004; Urich and Petersen, 2008; Soto et al., 2012). The media preparation, inoculation densities, incubation temperature, quality control organism, and interpretation of results were performed in compliance with the standards of the Clinical and (Clinical and Laboratory Standards Institute, 2014a). Briefly, the *Fno* isolates and *E. coli* ATCC 25922 were grown on agar as previously described and colonies suspended in sterile PBS to McFarland standard 0.5. This suspension was diluted 100-fold (*Fno*) or 1,000-fold (*E.coli* ATCC25922) in MMH and 50 μl of these added with a multichannel pipette to each well of the Sensititre® Plates. The plates were then incubated at 28°C and bacterial growth visually checked at 48 (*Fno* isolates) or 24 (*E. coli* ATCC 25922) hpi. The MIC value was defined as the lowest concentration with no visible growth.

#### Disc diffusion method

The susceptibility or resistance of *Fno* to 16 different antibiotics was investigated using the disc diffusion method on agar plates following the protocol established by the Clinical and Laboratory Standards Institute (2006) and (Soto et al., 2012) Briefly, bacteria were harvested after incubation in CHAH as previously described and suspended in PBS to achieve a turbidity equivalent to McFarland standard 0.5. Fresh CHAH plates were inoculated with 100 μl of the suspension using sterile disposable L shaped spreader. After 60 min when the plates had dried, antibiotic discs (Oxoid) were dispensed using a self-tamping antimicrobial susceptibility disc dispenser (Oxoid). Plates were incubated at 28°C for 96 h and the diameter of inhibition zones measured after 72 h.

### Genetic characterization (phylogenetic analyses)

Genomic DNA was obtained from STIR-GUS-F2f7 as previously described. The purity and concentration of the crude DNA was assessed from the 260/280 and 260/230 ratios obtained using a NanoDrop™ ND1000 (ThermoScientific, Delaware, USA) spectrophotometer.

Initially 12 housekeeping and core genes were selected for amplification and sequencing: 16S rRNA, 16S rRNA-23S rRNA intergenic spacer (ITS), 23S rRNA, malate dehydrogenase (*mdh*), chromosomal replication initiator protein alpha subunit (*dnaA*), DNA mismatch repair protein (*mutS*), phospho-glucomutase (*pgm*), peptide chain release factor 2 beta subunit (*prfB*), bifunctional proline dehydrogenase/pyrroline-5-carboxylate dehydrogenase alpha subunit (*putA*), DNA-directed RNA polymerase alpha subunit (*rpoA*), DNA-directed RNA polymerase beta subunit (*rpoB*), and triose-phosphate isomerase alpha subunit (*tpiA*). The suitability of these genes for phylogenetic analyses of *Francisella* spp. recovered from farmed aquatic organisms had been previusly reported by (Bohle et al., 2009; Ottem et al., 2009; Brevik et al., 2011).

In order to amplify the full length of the 12 genes from STIR-GUS-F2f7, 18 pairs of primers were designed based on the complete genome sequence of *Fno* Toba04, GenBank® accession number NC_017909.1 using Primer3 software (Untergasser et al., 2012). The primers were *in silico* tested using http://insilico.ehu.es/ and their attributes are presented in Supplementary Table 1.

PCR amplifications were performed using the ready to use 2x MyTaq™ HS Mix, (Bioline, London, UK), each reaction contained 25 μl of the mix, 1.0 μl of both forward and reverse primers (20 μM), 200 ng of the DNA template (~4 μl) and ultrapure water to a total volume of 50 μl. Cycling conditions consisted of an initial denaturation step of 1 min at 95°C, followed by 35 cycles of: 15 s at 95°C, 15 s at 66°C, and 10 s at 72°C performed in a Biometra TGradient Thermocycler (Biometra, Göttingen, Germany).

Amplification products were visualized on a 1% agarose gel stained with ethidium bromide. PCR products were purified for sequencing with the QIAquick PCR Purification Kit (QiaGen, California, USA) as directed by the manufacturer's instructions and sent for Sanger sequencing to GATC Biotech (GATC Biotech, Cologne, Germany). Of the 18 pairs of primers tested, 17 yielded products of the expected size (Supplementary Figure 3). No product was produced for *pgm* and this gene was therefore not further studied. The quality of the resulting chromatograms was visually checked using BioEdit® software version 7.1.11 and forward and reverse sequences assembled using the Multiple Sequence Comparison by Log-Expectation (MUSCLE) application of the MEGA (Molecular Evolutionary Genetic Analyses) package version 6 (Tamura et al., 2013). Consensus sequences were deposited in GenBank® with the accession numbers shown in Table 2.

**Table 2:** The GenBank accession number and final length of the sequenced genes from STIR-GUS-F2f7.

**Gene**	**Accession number**	**Length (bp)**
*dnaA*	KP657905	1,331
*mutS*	KP657899	2,429
*prfB*	KP657900	991
*putA*	KP657901	3,929
*rpoA*	KP657902	852
*rpoB*	KP657903	3,900
*tpiA*	KP657904	651
*mdh*	KP657898	696
16S rRNA+ITS+23S rRNA	KP657897	2,679

For each gene, the most similar sequences available from members of the genus *Francisella* were retrieved from GenBank® using the BLASTN® programs (Zhang et al., 2000) and aligned using the MUSCLE application of the MEGA software version 6 (Tamura et al., 2013). The NCBI accession number of all the individual sequences was indicated in the alignments. Furthermore, the gene sequences corresponding to each strain were concatenated using an in-house script developed with the programing language Perl available at https://www.perl.org/.

In addition, the partial 16S rRNA gene (1,425 bp) of STIR-GUS-F2F7 was compared with homologous sequences from other members of the family *Francisellaceae* including genera, species and subspecies that are currently described as “valid” in compliance with the International Code of Nomenclature of Prokaryotes and the International Committee on Systematics of Prokaryotes (Parte, 2013). In this alignment, the fish pathogens *Edwardsiella piscicida* C07-087 and *Piscirickettsia salmonis* AL10015 were included as outgroups.

All the alignments were manually adjusted and trimmed and their suitability for phylogenetic analyses double checked by computing the pairwise and the overall mean distances in MEGA package version 6 (Tamura et al., 2013). The 10 alignments were used to build phylogenetic trees and analyze the evolutionary relationship of *Fno* STIR-GUS-F2f7 with its closest members in the genus.

The evolutionary analyses were constructed in MEGA software version 6 (Tamura et al., 2013) using the Maximum Likelihood (ML) approach with exclusion of gaps and missing data. The model for each tree was chosen based on the best combination of model and rates among sites, and such combination was investigated for each alignment using the default settings of the “find best DNA/protein model” option. The reliability (reproducibility) of the trees was tested using the bootstrap method with 1,000 replications. In all analyses the nearest-neighbor-interchange was chosen as the ML heuristic method.

### Experimental infections

To fulfill Koch's postulates, 16 healthy naïve (from the TA, IoA, UoS) wild type and 16 red Nile tilapia fingerlings, 6–7 months/~11 g (7–13 g), were intraperitoneally (IP) injected with 0.1 ml of a suspension of STIR-GUS-F2f7 at an OD_600_ of 0.4 (~1.0 × 10^9^ CFU/ml). Due to the nature of the experiment and to comply with the local animal welfare regulations no replicate or control tanks were used. To perform the infections, the fish were moved in plastic bags into a flow-through system in the Aquatic Research Facility (ARF), IoA, UoS, where they were acclimated at 23 ± 2°C for 10 days prior to the infection trial. During acclimation and challenge periods, fish were kept at a stocking density of 8 fish per liter in 2 l plastic tanks and fed twice a day at a rate of 2% biomass. Prior to injection fish were anesthetized with TPQ (Pharmaq, Hampshire, UK) as previously described. Mortalities were monitored and recorded at least 4 times per day. Fish showing clinical signs were sampled for bacteriology and histopathology, pure cultures were obtained on CHA and they were confirmed as *Fno* by colony morphology and PCR. This study was carried out in accordance with the UK Animal (Scientific Procedures) Act 1986 and the University of Stirling Animal Welfare and Ethical Review Body (AWERB) regulations. All the relevant protocols were approved by the University of Stirling AWERB.

## Results

### Clinical samples and associated diagnostics

#### Post mortem examination

**Figure 1:**
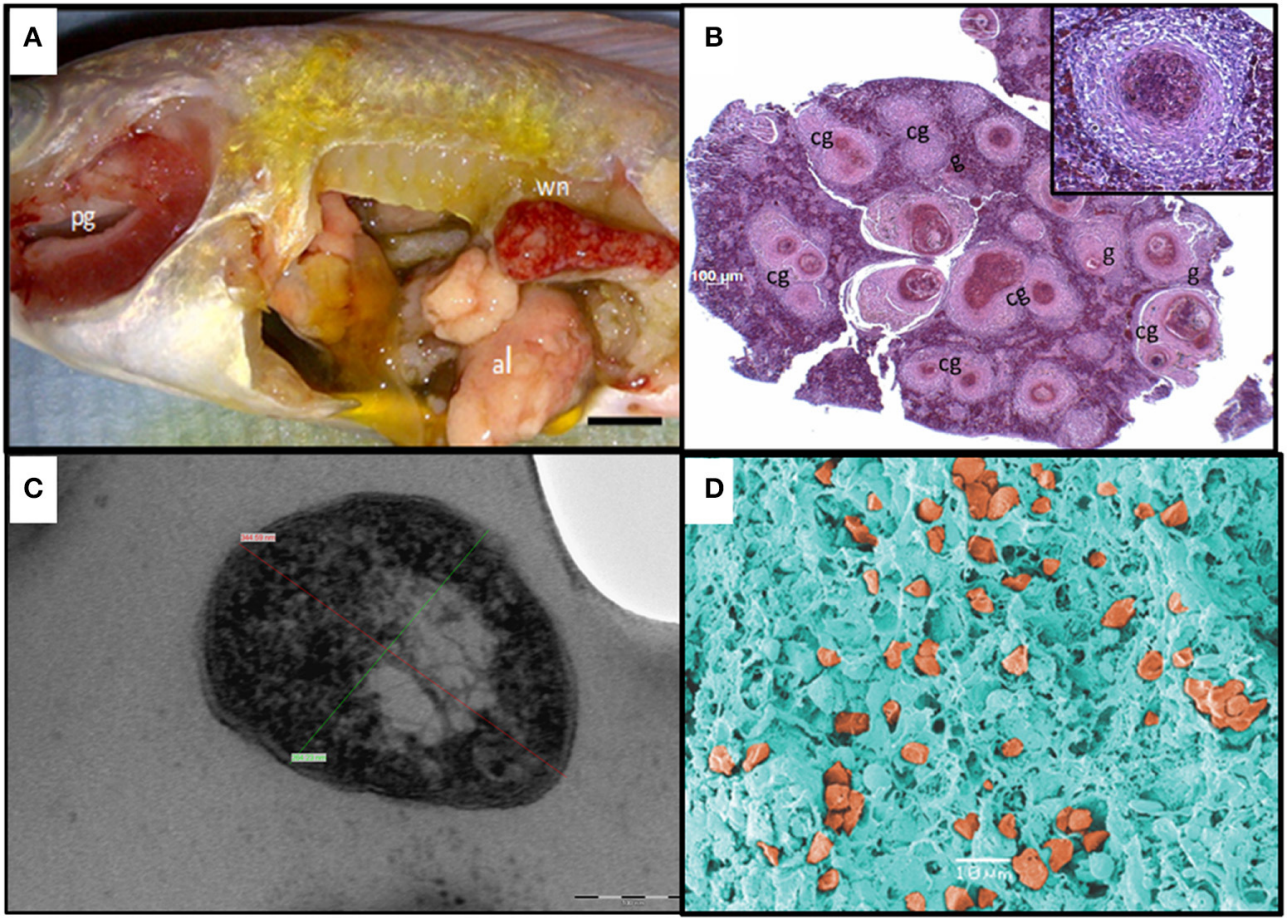
Diagnostics of piscine francisellosis. **(A)** Diseased red Nile tilapia (*Oreochromis niloticus*). wn, white nodules in the enlarged spleen; pg, pale gills; al, atrophic liver. **(B)** Histopathological cross section of the spleen shown in (A), using haematoxylin and eosin stain. g, granulomas; cg, coalescence of granulomas (40x magnification). Inset: granuloma (200x magnification). **(C)** Transmission electron micrograph of *Francisella*-like cell. Cell size: 344.59 nm (red line) × 264.23 nm (green line), scale bar = 100 nm. **(D)** Scanning electron micrograph showing several *Francisella*-like structures (orange) in the spleen.

The gross pathology displayed by the diseased fish included fin erosion, scale loss, pale skin, white gills, and emaciation. At necropsy, most internal organs were enlarged and either haemorrhagic or pale. In some fish, the anterior kidneys were hyperaemic and enlarged, resembling a raspberry in appearance. Most of the fish presented white nodules in the spleen, posterior kidney, and liver, in some severe cases covering over 60% of the organ surface (Figure 1A).

#### Histopathological, transmission (TEM), and scanning electron microscopy (SEM) analyses

Histological observations revealed the presence of extensive diffuse granulomatous inflammation. The affected tissues showed necrotising vasculitis and infiltration of mononuclear cells and neutrophils. The granuloma content was dominated by hypertrophied macrophages, fibroblasts, and leukocytes. Most granulomas exhibited necrotic cores. The most severely affected tissues were the spleen and the anterior kidney, with granuloma structures comprising up to 60% of the parenchyma. Granuloma formation was also seen in the heart, liver, and gills (Figure 1B).

**Figure 2:**
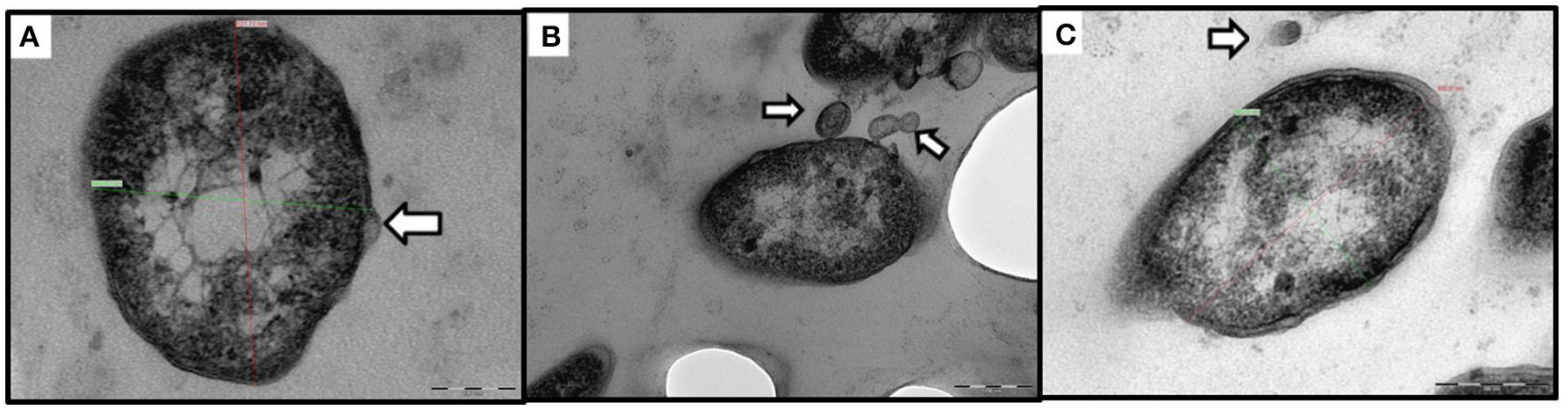
Transmission electron micrographs of outer membrane vesicles (OMV) like structures. **(A–C)** Extracellular localization of *Francisella*-like cells, the white arrows indicate OMV-like structures. **(A)** Bacterial membrane blebbing (scale bar = 100 nm). **(B)** Budding and secretion of vesicular membranous bodies (scale bar = 200 nm). **(C)** Bacterial cell next to a free floating vesicle (scale bar = 200 nm).

By TEM, pleomorphic coccobacillary bodies ranging in size from 0.2–0.4 μm (width) to 0.4–1.7 μm (length) were observed (Figure 1C). These structures could be observed inside phagocytic cells, free in the cytoplasm and most frequently within vacuoles surrounded by an electron lucent membrane. In SEM micrographs of spleen and head kidney the 3 dimensional structure of the bacterial cells resembled the shape of a corn grain (Figure 1D). Additionally, vesicles with electron dense membranes detaching from the bacterial cells were also detected by TEM. The size of these structures was 60–80 nm (width) 90–100 nm (length) (Figure 2).

#### Molecular diagnosis with a genus specific PCR

The Francisella genus specific PCR yielded a product of ~1.2 Kbp in *five* of the 10 samples tested from July 2012 confirming the presence of the bacteria within the fish tissues. In the samples recovered during the follow up visit in November 2012, the PCR analyses confirmed as positive all the fish from which it was possible to isolate the bacteria and also detected as positive four fish from where the isolation was not achieved. Interestingly the three biggest individuals, *two* of them the wild type Nile tilapia, were negative for both PCR and primary isolation (Table 3). In some fish, the results were not always the same for both tissues, in total *four* weak bands were observed in the agarose gel but were considered as positive after comparison with the controls (Supplementary Figure 1).

**Table 3:** Screening of red Nile tilapia sampled during follow up visit.

**Farm and fish**	***Fno* isolation**	**PCR**		**Total length of the fish (cms)**
		**Spleen**	**Kidney**	
Farm 1 fish 1	−	+	+	8.5
Farm 1 fish 2	−	−	+	6.5
Farm 1 fish 3	+	+	+	7.5
Farm 1 fish 4	+	+	+	6.0
Farm 1 fish 5	+	+	W	7.0
Farm 1 fish 6	+	+	+	6.5
Farm 1 fish 7	+	W	W	6.5
Farm 1 fish 8	−	−	−	6.0
Farm 1 fish 9	+	+	+	6.5
Farm 1 fish 10	+	+	−	6.0
Farm 2 fish 1	−	−	−	9.0
Farm 2 fish 2	−	−	−	22^*^
Farm 2 fish 3	−	−	−	20.5^*^
Farm 2 fish 4	−	+	−	18.5
Farm 2 fish 5	−	−	−	22
Farm 2 fish 6	−-	+	−	15
Farm 2 fish 7	+	+	+	9.5
Farm 2 fish 8	−	−	−	8.0
Farm 2 fish 9	+	W	−	8.5
Farm 2 fish 10	+	+	W	9.0

* *Wild type Nile tilapia; W, weak bands*.

### Bacterial isolation and identification

Of the different culture media tested, CHAH, CHATB and the commercial MTMA were able to support the primary recovery of *Fno*. No differences were observed between the CHATB plates with and without antibiotics, and no dominant or notable colonies were observed on TSA. The commercial MMLA failed to support bacterial growth directly from fish tissues. After molecular identification and purification, a total of 10 *Fno* isolates (seven from Farm 1 and three from Farm 2) were recovered and preserved (Table 1). The results obtained using all the different media and the spleen homogenates of fish 9 from Farm 2 (STIR-AVU-F2f9) are presented in Supplementary Figure 2.

### Phenotypic characterization

The optimal culture temperature *in vitro* was 28.0–28.5°C on agar plates for all *Fno* isolates tested, at this temperature the colonies appeared after 64 h. No growth was observed at temperatures of 18°C or lower or at 33°C or higher. Visible colonies appeared after 120 h at 22°C, 87 h at 24°C, and 69 h at 26°C. Although growth started to appear after only 48 h on plates incubated above 28°C, individual colonies on these plates were only visible after 72 h at 29°C, 75 h at 30°C, and 144 h at 32°C. Under the conditions described, the exponential phase of growth started after 15 h, the mid log phase was between 18 and 23 h and the stationary phase was reached after 30 h.

### Carbohydrate fermentation and enzymatic activity

Using the API20E kit, only the CIT (citrate utilization), VP (Voges–Proskauer reaction) and GEL (gelatinase) cups showed a positive reaction. This demonstrated the ability of the strains to utilize citrate as a carbon source, produce acetoin from sodium pyruvate and hydrolyse gelatine. No differences were observed between the novel *Fno* isolates and the type strain Ehime-1.

The use of the API ZYM kit revealed an identical profile among the different *Fno* isolates where eight of the 20 enzymes were reactive. These enzymes are (in decreasing order of intensity): acid phosphatase, naphthol-AS-BI-phosphohydrolase, esterase lipase (C8), alkaline phosphatase, esterase (C4), lipase (C14), α-chymotrypsin, and β-galactosidase.

### Carbon metabolism (metabolic fingerprint)

According to the inoculum gradient, an OD_600_ of 0.86 was found optimal for testing the *Fno* isolates in Biolog GN2 microplates. No differences were observed between the metabolic fingerprints of the isolates recovered from tilapia in the present study and the type strain Ehime-1 recovered from farmed grunt (Isaki) fish in Japan. The isolate PQ1104 from Costa Rica had an almost identical profile to the other *Fno* isolates with only 1 difference in the 95 carbon sources (i.e., acetic acid). The phenotypic fingerprints, excluding carbon sources that were negative for all, are presented in Table 4.

**Table 4:** Metabolic fingerprint of the different *Fno* isolates at an OD_600_ of 0.86.

**Carbon source test**		***Fno*** **0.86**			
**Well**		**1**	**2**	**3**	**4**
A1	Water	−	−	−	−
A3	Dextrin	+	+	+	+
A8	N-Acetyl-Dglucosamine	+	+	+	+
B2	D-Fructose	+	+	+	+
B6	α-D-Glucose	+	+	+	+
B12	D-Mannose	+	+	+	+
C11	Methyl Pyruvate	+	+	+	+
D1	Acetic Acid	+	+	−	+
E3	α-Keto Butyric Acid	+	+	+	+
F4	L-Alaninamide	+	+	+	+
F6	L-Alanine	+	+	+	+
F7	L-Alanylglycine	+	+	+	+
F8	L-Asparagine	+	+	+	+
F10	L-Glutamic Acid	+	+	+	+
G6	L-Proline	+	+	+	+
G9	L-Serine	+	+	+	+
G10	L-Threonine	+	+	+	+
H2	Inosine	+	+	+	+
H3	Uridine	+	+	+	+
H9	Glycerol	+	+	+	+
H10	D,L-α-Glycerol Phosphate	+	+	+	+
H11	Glucose-1-Phosphate	+	+	+	+
H12	Glucose-6-Phosphate	+	+	+	+

*Isolates: 1, Ehime-1; 2, STIR-GUS-F2f7; 3 PQ1104; 4, STIR-MATT-F1f6. Carbon sources that were negative for all the isolates are not presented*.

### Cellular fatty acid methyl esters profiles

The FAME profiles were similar for all *Fno* isolates tested and no differences in the order of the major components among the isolates were observed. The predominant fatty acids for isolate STIR-GUS-F2f7 were: 24:1 (20.3%), 18:1n-9 (16.9%), 24:0 (13.1%) 14:0 (10.9%), 22:0 (7.8%), 16:0 (7.6%), and 18:0 (5.5). An overview of the relative composition of the *Fno* isolates analyzed is presented in Table 5.

**Table 5:** Relative fatty acid composition (%) of STIR-GUS-F2f7 and the other *Fno* isolates after 43 h incubation in MMHB.

**Fatty acid**	**STIR-MATT-F1f6**	**STIR-GUS-F2f7**	**EHIME-1**	***Fno***
				**average**
14:0	10.96 ± 0.03	10.92 ± 0.30	9.72 ± 0.52	11
Iso 15:0	0.03 ± 0.03	0.06 ± 0.04	0.13 ± 0.03	0
15:0	0.27 ± 0.05	0.35 ± 0.05	0.37 ± 0.02	0
16:0	7 ± 0.18	8 ± 0.51	8 ± 0.12	8
17:0	0.45 ± 0.01	0.52 ± 0.04	0.54 ± 0.03	1
18:0	6.03 ± 0.09	5.50 ± 0.20	5.26 ± 0.12	6
19:0	0.17 ± 0.01	0.19 ± 0.01	0.25 ± 0.01	0
20:0	4.01 ± 0.10	3.61 ± 0.27	3.50 ± 0.07	4
16:0 3-OH	0.37 ± 0.05	0.52 ± 0.01	0.49 ± 0.02	0
21:0	0.09 ± 0.00	0.10 ± 0.01	0.15 ± 0.01	0
17:0 3-OH	0.18 ± 0.03	0.22 ± 0.09	0.39 ± 0.00	0
22:0	8 ± 0.09	8 ± 0.35	8 ± 0.06	8
18:0 3-OH	1.95 ± 0.22	2.67 ± 0.16	2.58 ± 0.16	2
23:0	0.42 ± 0.01	0.50 ± 0.04	0.63 ± 0.05	1
24:0	13 ± 1.38	13 ± 1.13	12 ± 0.09	13
25:0	0.00 ± 0.00	0.00 ± 0.00	0.00 ± 0.00	0
26:0	0.00 ± 0.00	0.00 ± 0.00	0.00 ± 0.00	0
Total saturated	53.61 ± 1.61	53.67 ± 1.22	51.39 ± 0.51	53
16:1n-9	0.29 ± 0.09	0.21 ± 0	0.24 ± 0.03	0
16:1n-7	0.54 ± 0.08	0.72 ± 0.18	0.66 ± 0.12	1
17:1	0.54 ± 0.02	0.78 ± 0.14	0.79 ± 0.05	1
18:1n-9	18 ± 0.28	17 ± 0.09	17 ± 0.31	17
18:1n-7	0.10 ± 0.06	0.12 ± 0.07	0.18 ± 0.01	0
19:1	0.17 ± 0.01	0.23 ± 0.02	0.24 ± 0.02	0
20:1	1.68 ± 0.03	1.63 ± 0.11	1.42 ± 0.03	2
21:1	0.01 ± 0.01	0.02 ± 0.02	0.00 ± 0.00	0
22:1n-11	3.55 ± 0.19	4.40 ± 0.83	3.93 ± 0.39	4
23:1	0.75 ± 0.02	0.98 ± 0.10	1.23 ± 0.07	1
24:1	21.0 ± 1.15	20 ± 0.82	23 ± 0.91	21
Total monounsaturated	46.32 ± 1.61	46.25 ± 1.21	48.46 ± 0.52	47
18:2n-6	0.07 ± 0.0	0.08 ± 0.01	0.29 ± 0.01	0
Total n-6 PUFA	0.07 ± 0	0.08 ± 0.01	0.29 ± 0.01	0
Total	100 ± 0	100 ± 0	100 ± 0	100

### Antibacterial susceptibility tests

#### Broth microdilution method

All the MICS for antibiotics which reference values at 28°C are available were within the range considered as acceptable by the Clinical and Laboratory Standards Institute (2014b), the lowest MIC values observed in the AVIAN1F Sensititre® plates across the replicates of the *Fno* tested were enrofloxacin (<0.12 μg/ml), gentamicin (<0.5 μg/ml), neomycin (<2 μg/ml), and streptomycin (<8 μg/ml) while the highest were ceftiofur (2 to >4 μg/ml), erythromycin (>4 μg/ml) sulphadimethoxine (128–256 μg/ml), trimethoprim/sulfamethoxazole (>2/38 μg/ml), penicillin (4 to >8 μg/ml), tylosin tartrate (20 to >20 μg/ml), and clindamycin (>4 μg/ml).

In the GN2F Sensititre® plates the lowest MIC values were amikacin (<8 μg/ml), ciprofloxacin (<0.5 μg/ml), gatifloxacin (<1 μg/ml), nitrofurantoin (<16 μg/ml), and tobramycin (<4 μg/ml) whereas the highest were aztreonam (16–32 μg/ml), cefazolin (32 to >32 μg/ml), cefotetan (>32 μg/ml), cefuroxime (32 to >32 μg/ml), and cefoxitin (>32 μg/ml). The range of variability observed in MIC values in both the AVIAN1F and the GN2F Sensititre® plates are summarized in Supplementary Table 2.

#### Disc diffusion method

In the disc diffusion method, 8 of the 16 antimicrobials developed clear and reproducible zones of inhibition among the *Fno* strains i.e., enrofloxacin (5 μg/disc), kanamycin (30 μg/disc), gentamicin (2 μg/disc), tetracycline (30 μg/disc), oxytetracycline (30 μg/disc), florfenicol (30 μg/disc), oxolinic acid (2 μg/disc), and streptomycin (10 μg/disc). No substantial differences were found amongst *Fno* isolates. The list of all the compounds tested and inhibition zone sizes (means and standard deviations) are presented in Supplementary Table 3.

### Genetic characterization (phylogenetic analyses)

When comparing the average nucleotide identity (ANI) the STIR-GUS-F2F7 gene sequences showed 99–100% resemblance with other *Fno* strains and after *Fno*, the closest related sequences were those belonging to members of *F. philomiragia* and *Fnn*, followed by the 4 *F. tularensis* subspecies, *F. halioticida* and *Allofrancisella guangzhouensis*. The similarity values (%) are presented in Supplementary Table 4.

**Figure 3:**
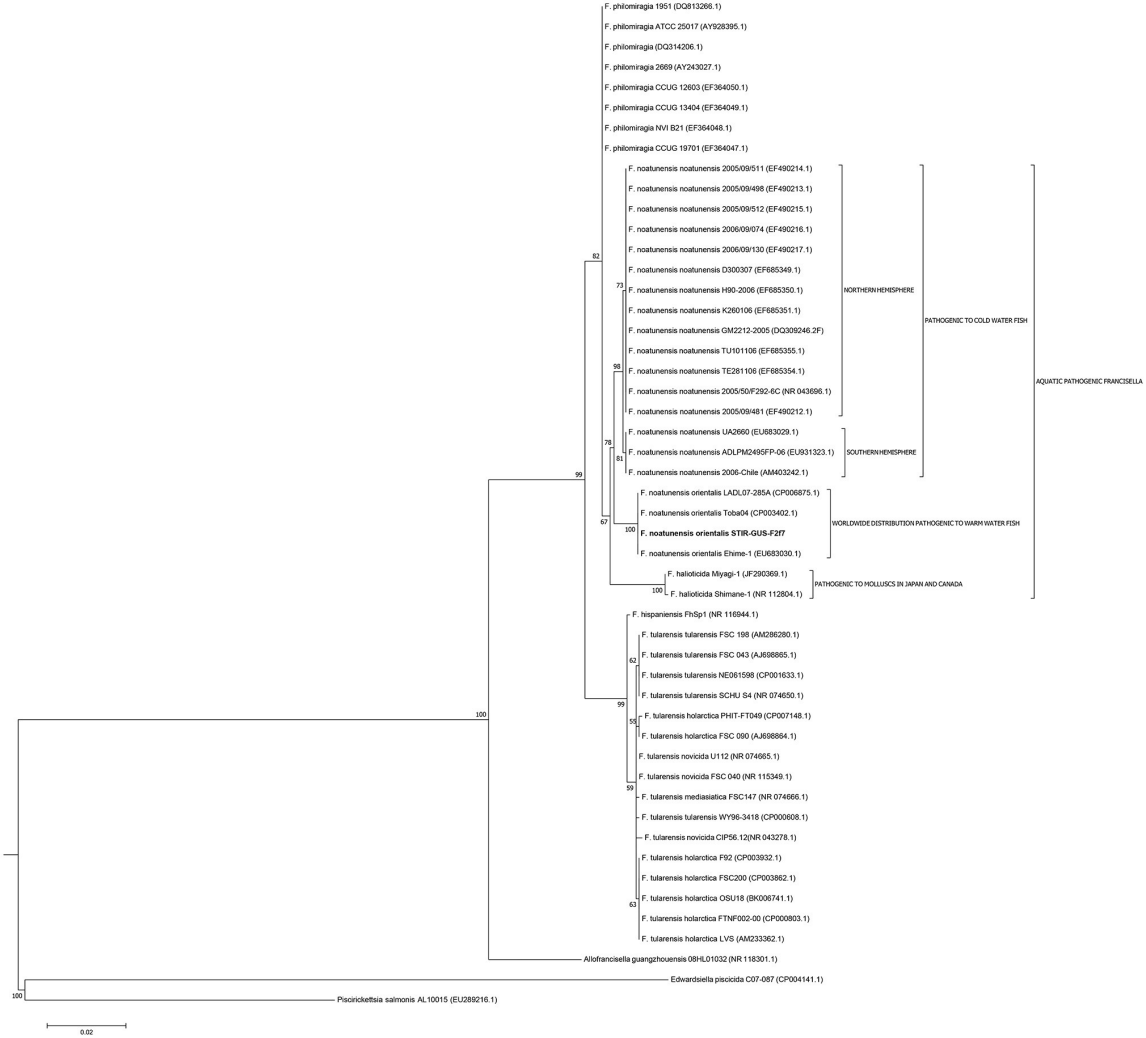
Molecular phylogenetic analysis of the family *Francisellaceae* based on 50 16S rRNA sequences (1,350 nt). The evolutionary history was inferred using the Maximum Likelihood method based on the Kimura 2-parameter model (Kimura, 1980). The tree with the highest log likelihood (−4004.5670) is shown. The percentage of trees in which the associated taxa clustered together is shown next to the branches. Initial trees for the heuristic search were obtained by applying the Neighbor-Joining method to a matrix of pairwise distances estimated using the Maximum Composite Likelihood (MCL) approach.

**Figure 4:**
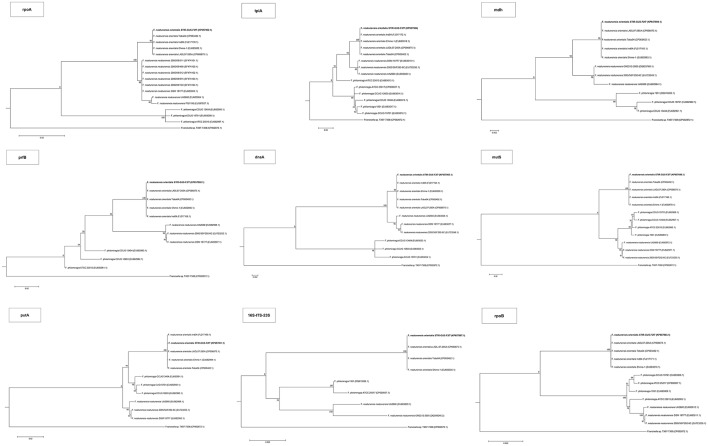
Maximum Likelihood trees for all the housekeeping gene sequences. The evolutionary history was inferred using the Maximum Likelihood method based on the model and rate differences among sites specified for each gene on Supplementary Table 5. The tree with the highest log likelihood is shown. The percentage of trees in which the associated taxa clustered together is shown next to the branches. In initial tree(s) for the heuristic search were obtained by applying the Neighbor-Joining method to a matrix of pairwise distances estimated using the Maximum Composite Likelihood (MCL) approach. The tree is drawn to scale, with branch lengths measured in the number of substitutions per site. All positions containing gaps and missing data were eliminated.

The 16S rRNA gene analysis (Figure 3) included sequences from validly described *Francisellaceae* species and subspecies and thus the phylogenetic tree illustrates the evolutionary history and the allocation of the new strains within the currently valid taxonomy of this family. The trees based on the core and housekeeping genes depict the evolutionary relationship of STIR-GUS-F2f7 with its closest related *taxa*: *Fnn* and *F. philomiragia* (Figure 4).

In all phylogenetic trees, the novel isolate STIR-GUS-F2f7 was seen to group within the *Fno* clade and in 9 of the 11 trees a subclade was observed within the *Fnn* isolates, this subdivision clustered strains recovered from cod in Norway and separated them from those isolated from Atlantic salmon farmed in Chile; this subgrouping was well supported with bootstrap values ranging from 70% in the 16S rRNA gene tree to 97% in the *mdh, putA*, and *rpoB* trees.

**Figure 5:**
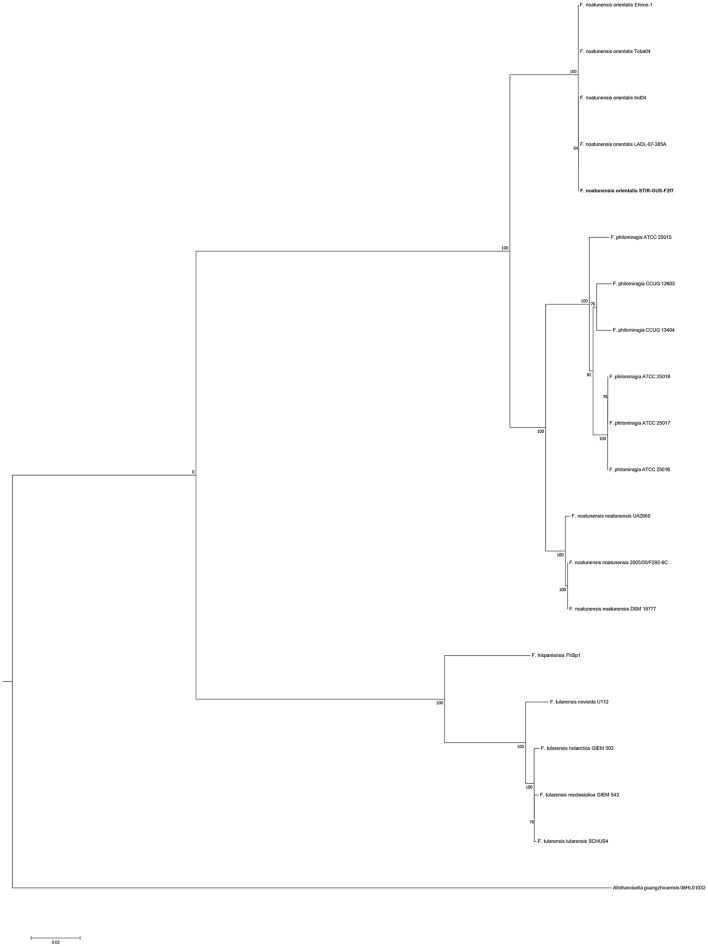
Molecular phylogenetic analysis of the family *Francisellaceae* based on the concatenated sequence of the house keeping genes (15,750 nt). The evolutionary history was inferred using the Maximum Likelihood method based on the General Time Reversible model (GTR+G+I). The tree with the highest log likelihood (−52,931.87) is shown. The percentage of trees in which the associated taxa clustered together is shown next to the branches. Initial trees for the heuristic search were obtained by applying the Neighbor-Joining method to a matrix of pairwise distances estimated using the Maximum Composite Likelihood (MCL) approach.

In the trees built with the short sequences i.e., *rpoA, tpiA, mdh, prfB, dnaA*, and 16S rRNA the *Fno* and *Fnn* appeared close to each other as a sibling taxa, however when longer sequences were used, including the concatenated sequence the evolutionary divergence between the two *Fn* subspecies seemed to be as deep, or even deeper than that between *Fno* and *F. philomiragia* (Figures 4, 5).

### Experimental infections

**Figure 6:**
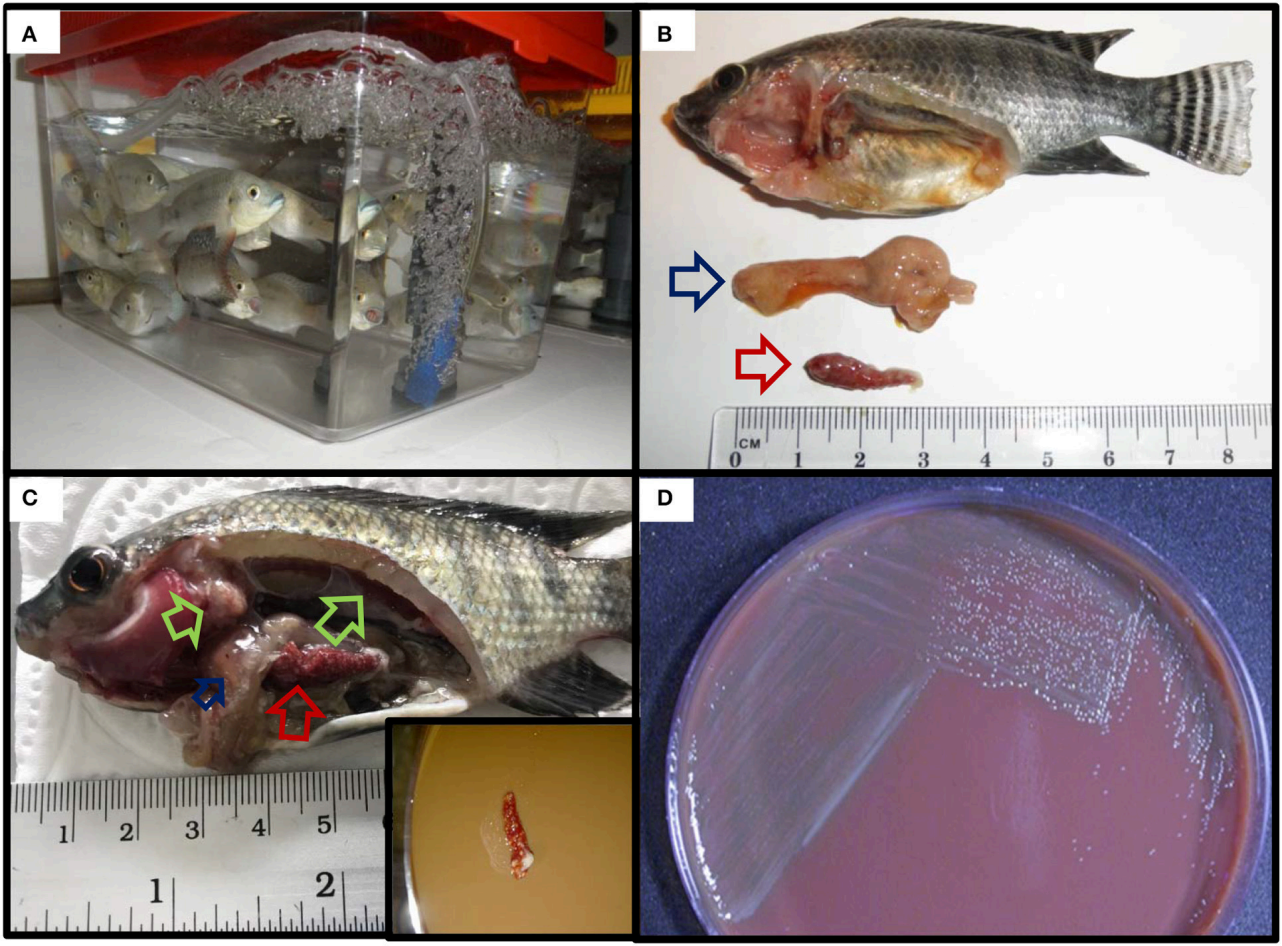
Fulfilment of Koch's postulates in Nile (wild type) tilapia. **(A)** Naïve tilapia fingerlings prior to the challenge. **(B,C)** Reproduction of clinical signs and gross lesions post challenge. Inset in **(C)**: streaking of spleen with widespread multifocal white nodules onto CHAH. **(D)** Recovery of pure colonies from the spleen of experimentally infected fish.

Koch's postulates were successfully fulfilled in both red and wild type Nile tilapia (Figure 6). All of the red tilapia died within the first 24–72 h showing signs of acute disease such as ascites and enlarged and haemorrhagic tissues. The wild type fish showed a less acute response with mortalities starting by day 2 and ending by day 5, the fish sampled at this stage showed pale gills, pale liver, and enlarged spleen and kidney with a more extended granuloma formation. Pure colonies of *Fno* (confirmed by colony morphology and PCR) were successfully isolated from the spleen of the clinically diseased fish.

## Discussion

During the last 10 years the application of molecular techniques has facilitated the identification of *Francisella* spp. causing severe mortalities in farmed fish. Unfortunately, despite the use of enriched commonly selective, media and established protocols the causative agent has not always been isolated and little or almost no information is available regarding the phenotypic properties of these pathogens.

In the present study, the anamnesis, gross pathology and histopathological findings observed during the field outbreaks were similar to previous reports of chronic francisellosis in tropical aquaculture (Birkbeck et al., 2011; Colquhoun and Duodu, 2011). This clearly warranted inclusion of this disease as a differential diagnosis, which subsequently led to isolation and comprehensive characterization of the pathogen.

The electron microscopy observations of heavily infected fish tissues allowed visualization of extracellular pleomorphic bacteria as well as cytoplasmic bound bacterial-like cells within macrophages. Interestingly, ultrastructures consistent with those recently described by Brudal et al. (2014) as “outer membrane vesicles” (OMVs) in *Fnn* grown *in vitro* and *in vivo* in zebrafish embryos were also observed secreted by STIR-GUS-Ff7 during infections in tilapia. If the vesicles observed contain virulence factors, they could have an application in the development of vaccines against francisellosis in warm water aquaculture therefore further research is required to characterize these OMVs like structures.

The PCR developed by Forsman et al. (1994) has the advantage of being cost effective, easy and rapid to perform, so it remains as a good option for diagnosis of francisellosis when the fish are showing clinical signs or internal lesions if economic and technical resources are limited.

However, since it cannot differentiate between *Francisella* species and cannot detect low numbers of bacterial cells a more sensitive and specific assay such as that recently proposed by Duodu et al. (2012) should be considered when detection of low bacterial loads is required.

In the present study it was possible to diagnose some fish from which isolation was not achieved but whether the larger fish sampled were asymptomatic carriers remains uncertain. Further research should focus on developing simple, specific, sensitive, and cost effective diagnostic tools that can detect asymptomatic carrier fish and can be applied at the farm level.

Of the culture medium tested, MMLA was included as an alternative to the MTMA which is not readily available by the manufacturer in Europe but it failed to support the primary isolation of *Fno* from fish despite having almost identical composition.

The use of tilapia blood in agars has been previously reported by Pasnik et al. (2005). In the present study the media containing cystine heart agar and tilapia blood (CHTB) with and without antibiotics proved successful for isolating and growing *Fno* and could be a useful in countries where the use of bovine by-products is banned for veterinary vaccine development.

The growth characteristics of the bacterium on CHAH and MMHB were the same for all *Fno* isolates tested including the reference strain Ehime-1, with growth from 21 to 33°C and an optimal *in vitro* growth temperature of 28–28.5°C. These results differ to reports by Ottem et al. (2009) where growth at 18°C was reported and 22°C was indicated as the optimal *in vitro* growth temperature for the type strain Ehime-1.

The API ZYM profiles were identical for all *Fno* isolates, and these results were similar to those previously reported for *Fno* by Ottem et al. (2009). These kits have the disadvantage that they are not designed for the characterization of fastidious bacteria and although some information was obtained from them they were not useful to observe differences between the *Fn* subspecies. The Biolog GN2 microplates do not depend upon bacterial growth as their chemistry is based on the reduction of tetrazolium, as a response to the metabolism of the carbon source rather than to metabolic by-products. There are no reports of the use of the Biolog GN2 microplates for characterization of *Fno*, but these plates have been used for the automated identification of *F. tularensis* (Whipp et al., 2003; Gyuranecz et al., 2010; Kreizinger et al., 2013) and characterization and description of *F. hispaniensis, Fnn* and *F. philomiragia* (Huber et al., 2010). Testing a gradient of inoculation densities revealed a number of positive reactions which would otherwise have been reported as negative using the inoculant concentrations recommended by the manufacturer.

As with the Biolog GN2 microplates, there are no previous reports of FAME profiling reported for any *Fno* isolate. The results obtained in this study for STIR-GUS-F2f7 and the other *Fno* isolates are consistent with the FAME signature of other species within the genus *Francisella* (Jantzen et al., 1979; Nichols et al., 1985). On comparison of the FAME profiles obtained for *Fno* in this study and those available for the closest members of the genus, *F. philomiragia* and *Fnn* (Ottem et al., 2007a,b; Huber et al., 2010), the most dissimilar groups are *Fno* and *F. philomiragia* with 8 fatty acids (24:1, 24:0, 22:0, 22:1n-11, 18:1n-9, 18:0, 16:0, and 20:0) having at least 3% of difference between them. Only two differences over 3% were seen between *Fnn* and *F philomiragia* in 22:0 and 18:0. The most divergent fatty acids between the *Fno* here reported and those available for *Fnn* in (Ottem et al., 2007a,b) are in 24:1, 18:1n-9, 24:0, 16:0, 14:0, 18:0. In order to investigate whether these dissimilarities are stable among these groups, further phenotypic studies using other techniques like, polar lipids, quinones, polyamines, metabolic profiles, antimicrobial susceptibility etc. should be performed with more *Fn* and the *F. philomiragia* type strain simultaneously.

In the broth microdilution test the results of the quality control validated the use of the Sensititre® plates with MMHB and this was in accordance with Soto et al. (2012) but inconsistent with Baker et al. (1985) for whom the use of the enrichments (IsoVitaleX™ and glucose) affected the results. The minor discrepancies observed between the present study and the MIC values obtained by Soto et al. (2012) in the range of 17 of the compounds may have been caused by the differences in the number of bacterial cells inoculated. In order to prevent this, it is proposed that the inoculum densities should be standardized at a specific OD_600_ with cells harvested at log phase rather than a McFarland standard.

Although there are no antibacterial clinical breakpoints established for *Fn*, it is suggested from the data observed in the Sensititre® Plates (distance between MIC and the lowest concentration tested) that the *Fno* strains analyzed are susceptible to enrofloxacin, gentamicin, neomycin, streptomycin, amikacin, ciprofloxacin, gatifloxacin, nitrofurantoin, and tobramycin.

As for the broth microdilution method, there are no established inhibition diameters for the classification of *Fno* using the disc diffusion method. However, based on diameters of the inhibition zones here observed, it can be interpreted that the isolates are susceptible to oxolinic acid, enrofloxacin, kanamycin, gentamicin, tetracycline, oxytetracycline, florfenicol, and streptomycin, this is the first time that kanamycin is tested on *Francisella* spp. recovered from fish. These observations are in agreement with Soto et al. (2012) who also tested enrofloxacin, gentamicin, tetracycline, and florfenicol with almost identical results.

The lack of inhibition zone development when testing penicillin G, amoxicillin, sulphamethoxazole/trimethoprim, lincomycin, oleandomycin, carbenicillin, polymyxin B, and ampicillin indicates resistance to these antibiotics, and this correlates with MIC assays where penicillin G, amoxicillin, and sulphamethoxazole/trimethoprim had very high MIC values.

Although oxolinic acid was not tested by the broth microdilution technique in the present study, the results here observed in the disc diffusion assays and previous research on *Fno* (Soto et al., 2012) and *Fnn* (Ottem et al., 2007a; Bohle et al., 2009; Isachsen et al., 2012) suggest that this compound should be comprised in future *Fn* antimicrobial resistance investigations including MIC determination.

Of the compounds that *in vitro* inhibited the growth of *Fno*, florfenicol, and oxytetracycline, appeared the best option to treat the fish as they are authorized for use in aquaculture in the European Union and previous reports have documented their kinetics in live fish suffering francisellosis (Soto et al., 2010, 2013; Colquhoun and Duodu, 2011).

Interestingly, the phylogenetic location of the *Fno* taxon was not consistent among the trees and the variation seemed associated with the length of the alignments i.e., the *Fno* clade appeared to be more distant from *Fnn* than *Fp* when sequences longer than 2,300 nucleotides were used. These results are in agreement with recent *Francisella* genomic studies where limited data from *Fno* and *Fnn* was included (Sjödin et al., 2012; Sridhar et al., 2012; Challacombe et al., 2017). In the context of the current taxonomy of this genus, these results suggest that *Fno* could represent a separate species however further research with more genomic data i.e., whole genome sequencing and phenotypic analyses of closely related taxa is needed.

## Conclusions

In conclusion the present study describes a set of tools that can be applied for the diagnosis of piscine francisellosis, describes the isolation of *Fno* from tilapia in Lincolnshire, England UK, and proposes a polyphasic approach for the characterization of this fastidious intracellular pathogen.

## Author contributions

Conception of the work: JGR-P, KT, RR, DP, and AA. Data collection: JGR-P and MM. Data analysis and interpretation: JGR-P, KS, ES, and DC. Drafting the article: JGR-P and ES. Critical revision of the article: KT, ES, RR, DP, DC, and AA. All the authors approved the final version of the article.

### Conflict of interest statement

The authors declare that the research was conducted in the absence of any commercial or financial relationships that could be construed as a potential conflict of interest.

## References

[B1] Assis G. B. N. Oliveira T. F. Gardner I. A. Figueiredo H. C. P. Leal C. A. G. (2017). Sensitivity and specificity of real-time PCR and bacteriological culture for francisellosis in farm-raised Nile tilapia (*Oreochromis niloticus* L.). J. Fish Dis. 40, 785–795. 10.1111/jfd.12559 27670740

[B2] Baker C. N. Hollis D. G. Thornsberry C. (1985). Antimicrobial susceptibility testing of *Francisella tularensis* with a modified Mueller-Hinton broth. J. Clin. Microbiol. 22, 212–215. 4031036 10.1128/jcm.22.2.212-215.1985 PMC268361

[B3] Berrada Z. L. Telford S. R. (2010). Diversity of *Francisella* species in environmental samples from Martha's Vineyard, Massachusetts. Microb. Ecol. 59, 277–283. 10.1007/s00248-009-9568-y 19669828 PMC2836248

[B4] Birkbeck T. H. Feist S. W. Verner-Jeffreys D. W. (2011). *Francisella* infections in fish and shellfish. J. Fish Dis. 34, 173–187. 10.1111/j.1365-2761.2010.01226.x 21306585

[B5] Bohle H. Tapia E. Martínez A. Rozas M. Figueroa A. Bustos P. (2009). *Francisella philomiragia*, a bacteria associated with high mortalities in Atlantic salmon (*Salmo salar*) cage farmed in Llanquihue lake. *Arch. Med*. Vet. 41, 237–244. 10.4067/S0301-732X2009000300008

[B6] Brevik Ø. J. Ottem K. F. Kamaishi T. Watanabe K. Nylund A. (2011). *Francisella halioticida* sp. nov., a pathogen of farmed giant abalone (*Haliotis gigantea*) in Japan. J. Appl. Microbiol. 111, 1044–1056. 10.1111/j.1365-2672.2011.05133.x 21883728

[B7] Brown S. D. Krisher K. Traczewski M. M. (2004). Broth microdilution susceptibility testing of *Francisella tularensis*: quality control limits for nine antimicrobial agents and three standard quality control strains. J. Clin. Microbiol. 42, 5877–5880. 10.1128/JCM.42.12.5877-5880.2004 15583330 PMC535293

[B8] Brudal E. Lampe E. O. Reubsaet L. Roos N. Hegna I. K. Thrane I. M.. (2014). Vaccination with outer membrane vesicles from *Francisella noatunensis* reduces development of francisellosis in a zebrafish model. Fish Shellfish Immunol. 42, 50–57. 10.1016/j.fsi.2014.10.025 25449706

[B9] Challacombe J. F. Petersen J. M. Hodge D. Pillai S. Kuske C. R. (2017). Whole-genome relationships among *Francisella* bacteria of diverse origins define new species and provide specific regions for detection. Appl. Environ. Microbiol. 83, e2589–e2516. 10.1128/AEM.02589-16 PMC5244304 27881415

[B10] Clinical and Laboratory Standards Institute (2006). Methods for Antimicrobial Disk Susceptibility Testing of Bacteria Isolated From Aquatic Animals: Approved Guideline. Clinical and Laboratory Standards Institute, Wayne, PA.

[B11] Clinical and Laboratory Standards Institute (2014a). Performance Standards for Antimicrobial Susceptibility Testing of Bacteria Isolated From Aquatic Animals; Second Informational supplement. Clinical and Laboratory Standards Institute, Wayne, PA.

[B12] Clinical and Laboratory Standards Institute (2014b). Methods for Broth Dilution Susceptibility Testing of Bacteria Isolated From Aquatic Animals; Approved Guideline- Second Edition. Clinical and Laboratory Standards Institute, Wayne, PA.

[B13] Colquhoun D. J. Duodu S. (2011). *Francisella* infections in farmed and wild aquatic organisms. Vet. Res. 42, 47–62. 10.1186/1297-9716-42-47 21385413 PMC3060124

[B14] Colquhoun D. J. Larsson P. Duodu S. Forsman M. (2014). Chapter 14 The family *Francisellaceae*, in The Prokaryotes, Gammaproteobacteria, 4th Edn., eds Rosenberg E. De Long E. F. Lory S. Stackebrandt E. Thompson F. (Berlin: Springer-Verlag), 287–314.

[B15] Cora M. C. Neel J. A. Tarigo J. Post K. Barnes J. (2010). *Francisella philomiragia* septicemia in a dog. J. Vet. Intern. Med. 24, 969–972. 10.1111/j.1939-1676.2010.0545.x 20649752

[B16] Duodu S. Larsson P. Sjödin A. Soto E. Forsman M. Colquhoun D. J. (2012). Real-time PCR assays targeting unique DNA sequences of fish-pathogenic *Francisella noatunensis* subspecies *noatunensis* and *orientalis*. Dis. Aquat. Org. 101, 225–234. 10.3354/dao02514 23324419

[B17] Ender P. T. Dolan M. J. (1997). Pneumonia associated with near-drowning. Clin. Infect. Dis. 25, 896–907. 9356805 10.1086/515532

[B18] Euzeby J. (2007). Notification that new names and new combinations have appeared in volume 57, part 9, of the IJSEM. Int. J. Syst. Evol. Microbiol. 57, 2727–2728. 10.1099/ijs0.65660-0 18319449

[B19] Euzeby J. (2008). List of new names and new combinations previously effectively, but not validly, published. Validation List No. 119. Int. J. Syst. Evol. Microbiol. 58, 1–2. 10.1099/ijs.0.65794-0 18319448

[B20] Euzeby J. (2009a). List of new names and new combinations previously effectively, but not validly, published. Validation List No. 128. Int. J. Syst. Evol. Microbiol. 59, 1555–1556. 10.1099/ijs.0.016253-0 18319448

[B21] Euzeby J. (2009b). Notification of changes in taxonomic opinion previously published outside the IJSEM. Int. J. Syst. Evol. Microbiol. 59, 1559–1560. 10.1099/ijs.0.016261-0 18599685

[B22] Forsman M. Sandstrom G. Sjöstedt A. (1994). Analysis of 16S Ribosomal DNA sequences of *Francisella* strains and utilization for determination of the phylogeny of the genus and for identification of strains by PCR. Int. J. Syst. Bacteriol. 44, 38–46. 10.1099/00207713-44-1-38 8123561

[B23] Friis-Møller A. Lemming L. E. Valerius N. H. Bruun B. (2004). Problems in identification of *Francisella philomiragia* associated with fatal bacteremia in a patient with chronic granulomatous disease. 1840-1842. J. Clin. Microbiol. 42, 1840–1842. 10.1128/JCM.42.4.1840-1842.2004 15071065 PMC387557

[B24] García del Blanco N. Marti C. G. De La Puente Redondo V. A. (2004). *In vitro* susceptibility of field isolates of *Francisella tularensis* subsp. *holarctica* recovered in Spain to several antimicrobial agents. Res. Vet. Sci. 76, 195–198. 10.1016/j.rvsc.2003.12.002 15046952

[B25] Gyuranecz M. Erdélyi K. Fodor L. Jánosi K. Szépe B. Füleki M.. (2010). Characterization of *Francisella tularensis* strains, comparing their carbon source utilization. Zoonoses Public Health 57, 417–422. 10.1111/j.1863-2378.2009.01238.x 19538455

[B26] Hollis D. G. Weaver R. E. Steigerwalt A. G. Wenger J. D. Moss C. W. Brenner D. J. (1989). *Francisella philomiragia* comb. nov. (formerly *Yersinia philomiragia*) and *Francisella tularensis* biogroup *novicida* (formerly *Francisella novicida*) associated with human disease. *J. Clin*. Microbiol. 27, 1601–1608. 10.1128/jcm.27.7.1601-1608.1989 PMC267622 2671019

[B27] Hsieh C. Y. Tung M. C. Tu C. Chang C. D. Tsai S. S. (2006). Enzootics of visceral granulomas associated with *Francisella*-like organism infection in tilapia (*Oreochromis* spp.). Aquaculture 254, 129–138. 10.1016/j.aquaculture.2006.03.044

[B28] Hsieh C. Y. Wu Z. B. Tung M. C. Tsai S. S. (2007). PCR and *in situ* hybridization for the detection and localization of a new pathogen *Francisella*-like bacterium (FLB) in ornamental cichlids. Dis. Aquat. Organ. 75, 29–36. 10.3354/dao075029 17523541

[B29] Huber B. Escudero R. Busse H. J. Seibold E. Scholz H. C. Anda P.. (2010). Description of *Francisella hispaniensis* sp. nov., isolated from human blood, reclassification of *Francisella novicida* (Larson et al., 1955) Olsufiev et al., 1959 as *Francisella tularensis* subsp. *novicida* comb. nov. and emended description of the genus *Francisella*. Int. J. Syst. Evol. Microbiol. 60, 1887–1896. 10.1099/ijs.0.015941-0 19783615

[B30] Isachsen C. H. Vågnes Ø. Jakobsen R. A. Samuelsen O. B. (2012). Antimicrobial susceptibility of *Francisella noatunensis* subsp. *noatunensis* strains isolated from Atlantic cod *Gadus morhua* in Norway. Dis. Aquat. Org. 98, 57–62. 10.3354/dao02430 22422129

[B31] Jantzen E. Berdal B. P. Omland T. (1979). Cellular fatty acid composition of *Francisella tularensis. J. Clin*. Microbiol. 10, 928–930. 10.1128/jcm.10.6.928-930.1979 PMC273296 521490

[B32] Jeffery K. R. Stone D. Feist S. W. Verner-Jeffreys D. W. (2010). An outbreak of disease caused by *Francisella* sp. *in* Nile tilapia *Oreochromis niloticus* at a recirculation fish farm in the UK. Dis. Aquat. Org. 91, 161–165. 10.3354/dao02260 21387995

[B33] Kimura M. (1980). A simple method for estimating evolutionary rates of base substitutions through comparative studies of nucleotide sequences. J. Mol. Evol. 16, 111–120. 10.1007/BF01731581 7463489

[B34] Kreitmann L. Terriou L. Launay D. Caspar Y. Courcol R. Maurin M.. (2015). Disseminated infection caused by *Francisella philomiragia*, France, 2014. Emerg. Infect. Dis. 21, 2260–2261. 10.3201/eid2112.150615 26583375 PMC4672438

[B35] Kreizinger Z. Makrai L. Helyes G. Magyar T. Erdélyi K. Gyuranecz M. (2013). Antimicrobial susceptibility of *Francisella tularensis* subsp. *holarctica* strains from Hungary, Central Europe. J. Antimicrob. Chemother. 68, 370–373. 10.1093/jac/dks399 23065699

[B36] Leal C. A. G. Tavares G. C. Figueiredo H. C. P. (2014). Outbreaks and genetic diversity of *Francisella noatunensis* subsp *orientalis* isolated from farm-raised Nile tilapia (*Oreochromis niloticus*) in Brazil. Genet. Mol. Res. 13, 5704–5712. 10.4238/2014.July.25.26 25117328

[B37] Lewisch E. Dressler A. Menanteau-Ledouble S. Saleh M. El-Matbouli M. (2014). Francisellosis in ornamental African cichlids in Austria. *Bull. Eur. Assoc*. Fish Pathol. 34, 63–70.

[B38] Lin Q. Li N. Fu X. Hu Q. Chang O. Liu L. (2015). An outbreak of granulomatous inflammation associated with *Francisella noatunensis* subsp. *orientalis* in farmed tilapia (*Oreochromis niloticus* × *O. aureus*) in China. Chin. J. Oceanol. Limnol. 34, 460–466. 10.1007/s00343-016-4311-2

[B39] Mailman T. Schmidt M. H. (2005). *Francisella philomiragia* adenitis and pulmonary nodules in a child with chronic granulomatous disease. Can. J. Infect. Dis. Med. Microbiol. 16, 245–248. 10.1155/2005/486417 18159552 PMC2095034

[B40] Mikalsen J. Colquhoun D. J. (2009). *Francisella asiatica* sp. nov. isolated from farmed Tilapia *(Oreochromis* spp*.)* and elevation of *Francisella philomiragia* subsp. *noatunensis* to species rank as *Francisella noatunensis* comb. nov., sp. nov. Int. J. Syst. Evol. Microbiol. [Epub ahead of print]. 10.1099/ijs.0.002139-0 19783606

[B41] Mikalsen J. Olsen A. B. Tengs T. Colquhoun D. J. (2007). *Francisella philomiragia* subsp. noatunensis subsp. nov., isolated from farmed Atlantic Cod (*Gadus morhua* L). Int. J. Syst. Evol. Microbiol. 57, 1960–1965. 10.1099/ijs.0.64765-0 17766855

[B42] Nguyen V. V. Dong H. T. Senapin S. Pirarat N. Rodkhum C. (2016). *Francisella noatunensis* subsp. orientalis, an emerging bacterial pathogen affecting cultured red tilapia (*Oreochromis* sp.) in Thailand. Aquacult. Res. 47, 3697–3702. 10.1111/are.12802

[B43] Nichols P. D. Mayberry W. R. Antworth C. P. White D. C. (1985). Determination of monounsaturated double-bond position and geometry in the cellular fatty acids of the pathogenic bacterium *Francisella tularensis. J. Clin*. Microbiol. 21, 738–740. 10.1128/jcm.21.5.738-740.1985 PMC271770 3998104

[B44] Ortega C. Mancera G. Enríquez R. Vargas A. Martínez S. Fajardo R.. (2016). First identification of *Francisella noatunensis* subsp. orientalis causing mortality in Mexican tilapia *Oreochromis* spp. Dis. Aquat. Org. 120, 205–215. 10.3354/dao02999 27503916

[B45] Ostland V. E. Stannard J. A. Creek J. J. Hedrick R. P. Ferguson H. W. Carlberg J. M.. (2006). Aquatic *Francisella*-like bacterium associated with mortality of intensively cultured hybrid striped bass *Morone Chrysops* x *M. Saxatilis*. Dis. Aquat. Org. 72, 135–145. 10.3354/dao072135 17140136

[B46] Ottem K. F. Nylund A. Isaksen T. E. Karlsbakk E. Bergh Ø. (2008). Occurrence of *Francisella piscicida* in farmed and wild Atlantic cod, *Gadus morhua* L., in Norway. J. Fish Dis. 31, 525–534. 10.1111/j.1365-2761.2008.00930.x 18482383

[B47] Ottem K. F. Nylund A. Karlsbakk E. Friis-Møller A. Kamaishi T. (2009). Elevation of *Francisella philomiragia* subsp. *noatunensis* Mikalsen et al. (2007) to *Francisella noatunensis* comb. nov. [syn. *Francisella piscicida* Ottem et al. (2008) syn. nov.] and characterization of *Francisella noatunensi*s subsp. *orientalis* subsp. nov., two important fish pathogens. J. Appl. Microbiol. 106, 1231–1243. 10.1111/j.1365-2672.2008.04092.x 19187160

[B48] Ottem K. F. Nylund A. Karlsbakk E. Friis-Møller A. Krossøy B. (2007a). Characterization of *Francisella* sp., GM2212, the first *Francisella* isolate from marine fish, Atlantic cod (*Gadus morhua*). Arch. Microbiol. 187, 343–350. 10.1007/s00203-006-0198-1 17160676

[B49] Ottem K. F. Nylund A. Karlsbakk E. Friis-Møller A. Krossøy B. Knappskog D. (2007b). New species in the genus Francisella (Gammaproteobacteria; Francisellaceae); *Francisella piscicida* sp. nov. isolated from cod (*Gadus morhua*). Arch. Microbiol. 188, 547–550. 10.1007/s00203-007-0274-1 17619856

[B50] Parte A. C. (2013). LPSN—list of prokaryotic names with standing in nomenclature. Nucleic Acids Res. 42, D613–D616 10.1093/nar/gkt1111 24243842 PMC3965054

[B51] Pasnik D. J. Evans J. J. Klesius P. H. (2005). Nile tilapia, *Oreochromis niloticus*, blood agar and the culture of fish bacterial pathogens. *Bull. Eur. Assoc*. Fish Pathol. 25, 221–227.

[B52] Relich R. F. Humphries R. M. Mattison H. R. Miles J. E. Simpson E. R. Corbett I. J. (2015). *Francisella philomiragia* bacteremia in a patient with acute respiratory insufficiency and acute-on-chronic kidney disease. J. Clin. Microbiol. 12, 3947–3950. 10.1128/JCM.01762-15 PMC4652090 26400786

[B53] Ruane N. M. Bolton-Warberg M. Rodger H. D. Colquhoun D. J. Geary M. McCleary S. J.. (2013). An outbreak of francisellosis in wild-caught Celtic sea Atlantic cod, *Gadus morhua* L., juveniles reared in captivity. J. Fish Dis. 38, 97102. 10.1111/jfd.12210 24261672

[B54] Seward R. J. Ehrenstein B. Grundmann H. J. Towner K. J. (1997). Direct comparison of two commercially available computer programs for analysing DNA fingerprinting gels. J. Med. Microbiol. 46, 314–320. 10.1099/00222615-46-4-314 9128195

[B55] Sjödin A. Svensson K. Öhrman C. Ahlinder J. Lindgren P. Duodu S.. (2012). Genome characterisation of the genus *Francisell*a reveals insight into similar evolutionary paths in pathogens of mammals and fish. BMC Genomics 13:268. 10.1186/1471-2164-13-268 22727144 PMC3485624

[B56] Soto E. Endris R. G. Hawke J. P. (2010). *In vitro* and *in vivo* efficacy of florfenicol for treatment of *Francisella asiatica* infection in tilapia. Antimicrob. Agents Chemother. 54, 4664–4670. 10.1128/AAC.00206-10 20713674 PMC2976172

[B57] Soto E. Griffin M. Wiles J. Hawke J. P. (2012). Genetic analysis and antimicrobial susceptibility of *Francisella noatunensis* subsp. *orientalis* (*syn. F. asiatica*) isolates from fish. Vet. Microbiol. 154, 407–412. 10.1016/j.vetmic.2011.07.030 21868177

[B58] Soto E. Hawke J. P. Fernandez D. Morales J. A. (2009). *Francisella* sp., an emerging pathogen of tilapia, *Oreochromis niloticus* (L.) in Costa Rica. J. Fish Dis. 32, 713–722. 10.1111/j.1365-2761.2009.01070.x 19515205

[B59] Soto E. Kidd S. Gaunt P. S. Endris R. (2013). Efficacy of florfenicol for control of mortality associated with *Francisella noatunensis* subsp. *orientalis* in Nile tilapia, *Oreochromis niloticus (L.)*. J. Fish Dis. 36, 411–418. 10.1111/j.1365-2761.2012.01425.x 23134104

[B60] Sridhar S. Sharma A. Kongshaug H. Nilsen F. Jonassen I. (2012). Whole genome sequencing of the fish pathogen *Francisella noatunensis* subsp. *orientalis* Toba04 gives novel insights into *Francisella* evolution and pathogenecity. BMC Genomics 13:598. 10.1186/1471-2164-13-598 23131096 PMC3532336

[B61] Tamura K. Stecher G. Peterson D. Filipski A. Kumar S. (2013). MEGA6: molecular evolutionary genetics analysis version 6.0. Mol. Biol. Evol. 30, 2725–2729. 10.1093/molbev/mst197 24132122 PMC3840312

[B62] Tocher D. R. Harvie D. G. (1988). Fatty Acid Compositions of the major phosphoglycerides from fish neural tissues; (n-3) and (n-6) polyunsaturated fatty acids in rainbow trout (*Salmo gairdneri*) and cod (*Gadus morhua*) brains and retinas. Fish Physiol. Biochem. 5, 229–239. 10.1007/BF01874800 24226784

[B63] Untergasser A. Cutcutache I. Koressaar T. Ye J. Faircloth B. C. Remm M.. (2012). Primer3 – new capabilities and interfaces. Nucleic Acids Res. 40, e115. 10.1093/nar/gks596 22730293 PMC3424584

[B64] Urich S. K. Petersen J. M. (2008). *In vitro* susceptibility of isolates of *Francisella tularensis* types A and B from North America. Antimicrob. Agents Chemother. 52, 2276–2278. 10.1128/AAC.01584-07 18411318 PMC2415761

[B65] Wenger J. D. Hollis D. G. Weaver R. E. Baker C. N. Brown G. R. Brenner D. J. (1989). Infection caused by *Francisella philomiragia* (formerly *Yersinia philomiragia*): a newly recognized human pathogen. Ann. Intern. Med. 11, 888–892. 10.7326/0003-4819-110-11-888 2541646

[B66] Whipp M. J. Davis J. M. Lum G. de Boer J. Zhou Y. Bearden S. W. (2003). Characterization of a *novicida*-like subspecies of *Francisella tularensis* isolated in Australia. *J. Med*. Microbiol. 52, 839–842. 10.1099/jmm.0.05245-0 12909664

[B67] Whitehouse C. A. Kesterson K. E. Duncan D. D. Eshoo M. W. Wolcott M. (2012). Identification and characterization of *Francisella* species from natural warm springs in Utah, USA. Lett. Appl. Microbiol. 54, 313–324. 10.1111/j.1472-765X.2012.03214.x 22283482

[B68] Zerihun M. A. Feist S. W. Bucke D. Olsen A. B. Tandstad N. M. Colquhoun D. J. (2011). Francisella noatunensis subsp. noatunensis is the aetiological agent of visceral granulomatosis in wild Atlantic cod *Gadus morhua. Dis. Aquat. Org*. 95, 65–67. 10.3354/dao02341 21797037

[B69] Zhang Z. Schwartz S. Wagner L. Miller W. (2000). A greedy algorithm for aligning DNA sequences. J. Comput. Biol. 7, 1–2. 10.1089/10665270050081478 10890397

