# Supplementary material for: A Polyphasic Approach for Phenotypic and Genetic Characterization of the Fastidious Aquatic Pathogen Francisella noatunensis subsp. orientalis
Source: Front Microbiol. 2017 Dec 12;8:2324. doi: 10.3389/fmicb.2017.02324 (PMC5733052; doi:10.3389/fmicb.2017.02324)
Supplement: Supplementary file 1 [file Image1.pdf]

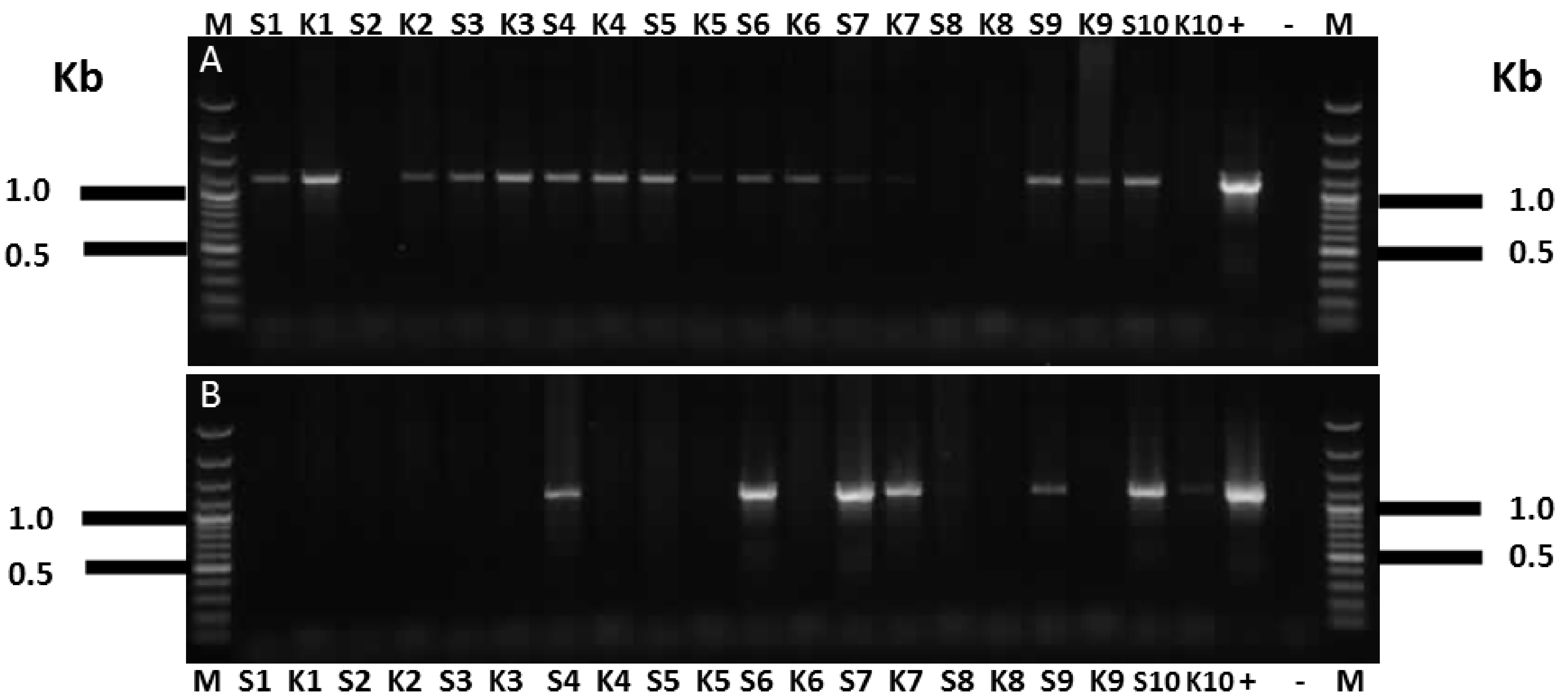

Supplementary Figure 1 Ethidium bromide stained 1% agarose gel after electrophoresis. Molecular confirmation by PCR of piscine francisellosis in samples recovered from a follow up visit to the farms. **A** representative gel of fish from Farm 1 and **B** representative gel of fish from Farm 2. **M=**100bp molecular weight markers. **S** =spleen and **K**=kidney. The numbers indicate the sampled fish. **+** Positive control. **–** Negative control.

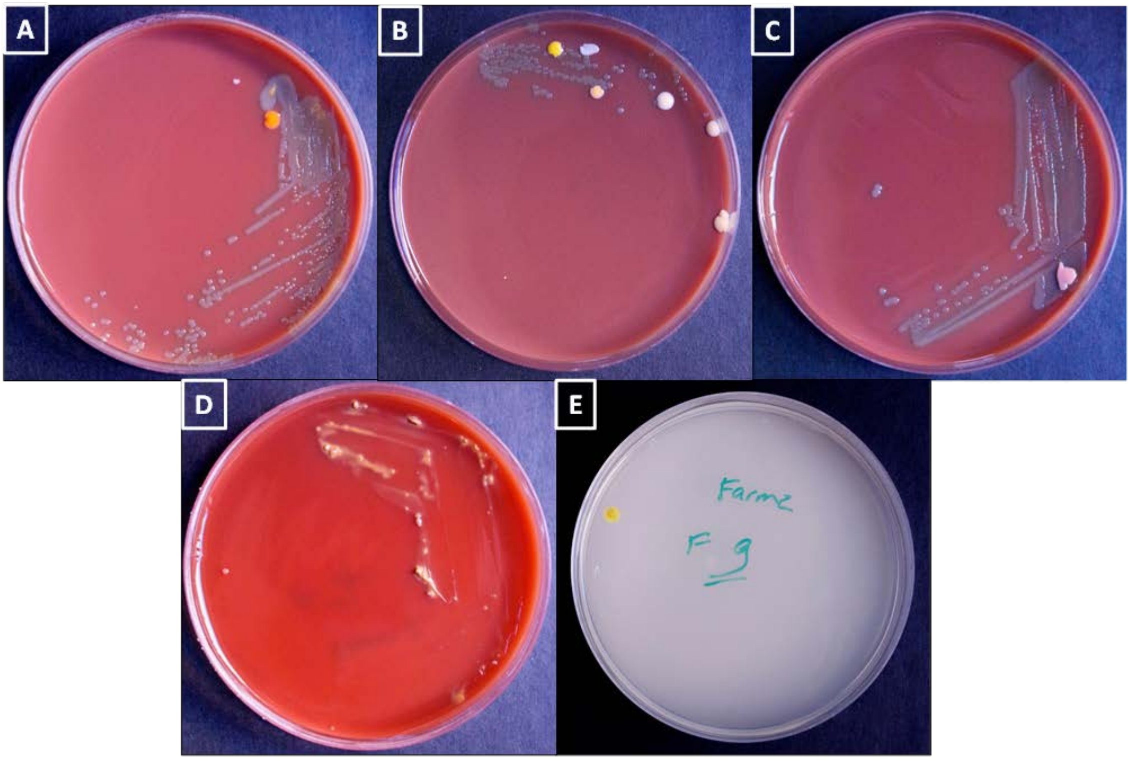

Supplementary Figure 2 Media used for the primary isolation of *Francisella* sp. from Nile tilapia. **A.** Cysteine heart agar + bovine haemoglobin. **B.** Modified Thayer Martin agar. **C.** Cysteine heart agar tilapia blood. **D.** Modified Martin Lewis agar. **E.** Trypticase soya agar. In media where it grew, the colonies were convex, smooth and with a grey-greenish colour**.**

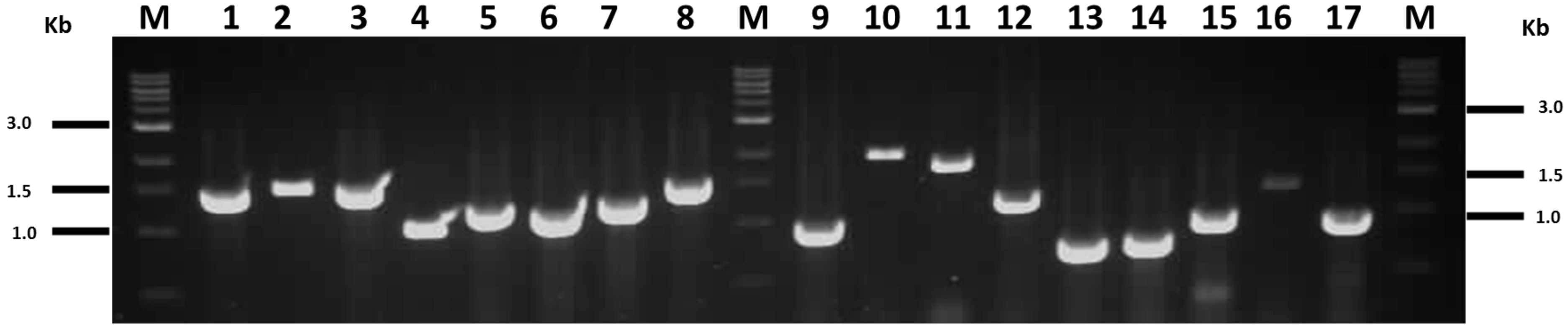

Supplementary Figure 3 Ethidium bromide stained 1% agarose gel after electrophoresis. PCR amplification of *Fno* STIR-GUS-F2f7 housekeeping genes. **M** 1Kb molecular markers. **1** *dnaA*. **2** *mutS*-1. **3** *mutS*-2. **4** *prfB*. **5** *putA*-1. **6** *putA*-2. **7** *putA*-3. **8** *putA*-4. **9** *rpoA*. **10** *rpoB*-1. **11** *rpoB*-2. **12** *rpoB*-3. **13** *tpiA*. **14** *mdh*. **15** 16SrRNA-ITS-23SrRNA-1. **16** 16SrRNA-ITS-23SrRNA-2. **17** 16SrRNA-ITS-23SrRNA-3.
