# Supplementary material for: A Polyphasic Approach for Phenotypic and Genetic Characterization of the Fastidious Aquatic Pathogen Francisella noatunensis subsp. orientalis
Source: Front Microbiol. 2017 Dec 12;8:2324. doi: 10.3389/fmicb.2017.02324 (PMC5733052; doi:10.3389/fmicb.2017.02324)
Supplement: Supplementary file 2 [file Table1.docx]

| **Supplementary Table 1** Overview of the primers designed for amplification and sequencing of selected housekeeping genes from STIR-GUS-F2f7 | | | | | | |
| --- | --- | --- | --- | --- | --- | --- |
| Gene short name | Primer | Sequence | Position in Toba04 | Tm °C | GC-Content % | Expected amplicon (bp) |
| *dnaA* | dnaA-F1 | AAAACCTTTCTACGTTTGAAT | 56-76 | 50.1 | 28.6 |  |
| *dnaA* | dnaA-R1 | GCAACTCATAATCATCAGAT | 1471-1452 | 51.2 | 35 | 1415 |
|  |  |  |  |  |  |  |
| *mutS* | mutS-F1 | TAGTTCAAAGAGTATATTTAG | 703305-703325 | 54.0 | 38.1 |  |
| *mutS* | mutS-R1 | TGACATTGAAAGGATAATTTCTC | 704814-704792 | 57.3 | 50 |  |
| *mutS* | mutS-F2 | GGCGTCTGATATACTCAGCT | 704375-704394 | 53.5 | 30.4 |  |
| *mutS* | mutS-R2 | AGAAGTTTCAAATCACACACC | 705831-705811 | 48.1 | 23.8 | 2526 |
|  |  |  |  |  |  |  |
| *pgm* | pgm-F1 | TGGCTATTCAGACTGTATCTAC | 1084092-1084113 | 56.5 | 40.9 |  |
| *pgm* | pgm-R1 | CAGTCATTCCTGTCAGAGAT | 1085705-1085686 | 55.3 | 45 | 1613 |
|  |  |  |  |  |  |  |
| *prfB* | prfB-F1 | TTGAATCTTTACGGGACTAT | 141503-141522 | 56.5 | 40.9 |  |
| *prfB* | prfB-R1 | AACTTATCTAAATCACCATCTA | 142512-142491 | 57.1 | 39.1 | 1009 |
|  |  |  |  |  |  |  |
| *putA* | putA-F1 | CTTATTGAACCATTCAGGTGAG | 1737813-1737834 | 56.5 | 40.9 |  |
| *putA* | putA-R1 | GCTTGGTAAGAAACATCTGTATG | 1738970-1738948 | 57.1 | 39.1 |  |
| *putA* | putA-F2 | AGAGCAAGGCTTAGCAGGTT | 1738905-1738924 | 57.3 | 50 |  |
| *putA* | putA-R2 | GAGTTTGTTCAGCAGGTTTA | 1739995-1739976 | 53.2 | 40 |  |
| *putA* | putA-F3 | GACTTCGCAGTGTTAGCAGA | 1739407-1739426 | 57.3 | 50 |  |
| *putA* | putA-R3 | GACCAAATTGTTCTCTACCAA | 1740613-1740593 | 54.0 | 38.1 |  |
| *putA* | putA-F4 | TGATTGTTGGTGCGATGAAAG | 1740371-1740391 | 55.9 | 42.9 |  |
| *putA* | putA-R4 | CGATAGTATCAACACTGACAG | 1741828-1741808 | 55.9 | 42.9 | 4015 |
|  |  |  |  |  |  |  |
| *rpoA* | rpoA-F1 | CAGGAATTTGTACCTAATATAC | 220361-220382 | 52.8 | 31.8 |  |
| *rpoA* | rpoA-R1 | TGAACACCTAGAGATAAGTTG | 221274-221254 | 54.0 | 38.1 | 913 |
|  |  |  |  |  |  |  |
| *rpoB* | rpoB-F1 | ATTCGCAAAGAGTTTGGGGT | 616228-616247 | 55.3 | 45 |  |
| *rpoB* | rpoB-R1 | GCTGAAACCATCTGCTTAGC | 618194-618175 | 57.3 | 50 |  |
| *rpoB* | rpoB-F2 | ATTCTTTACATCTGGTGCTT | 617718 -617737 | 51.2 | 35 |  |
| *rpoB* | rpoB-R2 | CTGGAGTACCATCTTCCATA | 619519-619500 | 55.3 | 45 |  |
| *rpoB* | rpoB-F3 | GGCGCGTAAAGATTTCGATG | 619068-619087 | 57.3 | 50 |  |
| *rpoB* | rpoB-R3 | TTCCTCAGATGAGTAGTCAA | 620268-620249 | 53.2 | 40 | 4040 |
|  |  |  |  |  |  |  |
| *tpiA* | tpiA-F1 | TGGGAAATTGGAAAATGAAT | 553453-553472 | 49.1 | 30 |  |
| *tpiA* | tpiA-R1 | CAATCAAGCCACCATCAAC | 554127-554109 | 54.5 | 47.4 | 674 |
|  |  |  |  |  |  |  |
| *mdh* | mdh-F1 | ACTTTCTGCCATTTGAATAC | 1769342-1769361 | 53.2 | 40 |  |
| *mdh* | mdh-R1 | GCTTATTGGTGCTGGTAATA | 1770056-1770037 | 52.1 | 35 | 714 |
|  |  |  |  |  |  |  |
| 16srRNA+ITS+23srRNA | 16-23s-F1 | GTTTCCCTTTCCACTGCG | 461307-461324 | 53.7 | 50 |  |
| 16srRNA+ITS+23srRNA | 16-23s-R1 | GTGTTAATCTGCGATAAG | 462231-462214 | 53.7 | 50 |  |
| 16srRNA+ITS+23srRNA | 16-23s-F2 | CACTTCGCTCGCCACTACTA | 462036- 462055 | 57.3 | 50 |  |
| 16srRNA+ITS+23srRNA | 16-23s-R2 | TGGTAGTCCACGCTGTAAAC | 463338-463319 | 59.4 | 55 |  |
| 16srRNA+ITS+23srRNA | 16-23s-F3 | ACCAGGTAAGGTTCTTCG | 463144-463161 | 49.1 | 38.9 |  |
| 16srRNA+ITS+23srRNA | 16-23s-R3 | AACGGTAACAGGTCTTCG | 464059-464042 | 56.00 | 55.60 | 2752 |
| The location of the primers is given in relation to the open reading frame (ORF) of the protein encoding and rRNA genes from the *Fno* reference genome Toba04, NCBI accession number [NC_017909](https://www.ncbi.nlm.nih.gov/nuccore/NC_017909) | | | | | | |
|  | | | | | | |

| **Supplementary Table 2** Minimal inhibitory concentration (MIC) of the *Francisella noatunensis orientalis* isolates and the quality control *Escherichia coli* ATTC 25922 by the broth microdilution method to 39 different antimicrobial compounds | | | | | | | | | | | |
| --- | --- | --- | --- | --- | --- | --- | --- | --- | --- | --- | --- |
|  | | | | | | | | | | | |
| Antimicrobial tested (concentration) | PQ1104 | | STIR-GUS-F2f7 | | Ehime-1 | | STIR-MATT-F1f6 | | *Fno* | *E. coli* ATCC 25922 | |
|  | 1 | 2 | 1 | 2 | 1 | 2 | 1 | 2 | range | present results | CLSI range |
| Enrofloxacin (2–0.12 mµ/ml) | <0.12 | <0.12 | 0.25 | <0.12 | <0.12 | <0.12 | <0.12 | <0.12 | <0.12 | <0.12 | 0.008-0.03 |
| Gentamycin (8–0.5 mµ/ml) | <0.5 | <0.5 | <0.5 | <0.5 | <0.5 | <0.5 | <0.5 | <0.5 | <0.5 | 0.5 | 0.25-1 |
| Ceftiofur (4–0.25 mµ/ml) | >4 | 4 | >4 | 2 | >4 | 4 | >4 | 4 | 2 to >4 | 0.5 | 0.25-1* |
| Neomycin (32–2 mµ/ml) | <2 | <2 | <2 | <2 | <2 | <2 | <2 | <2 | <2 | 8 | N/A |
| Erythromycin (4–0.25 mµ/ml) | >4 | >4 | >4 | >4 | >4 | >4 | >4 | >4 | >4 | >4 | N/A |
| Oxytetracycline (8–0.5 mµ/ml) | 4 | 0.5 | 4 | <0.25 | 1 | 0.5 | 2 | 0.5 | <0.25 to 4 | 2 | 0.5-2 |
| Tetracycline (8–0.25 mµ/ml) | 4 | 0.5 | 4 | 0.25 | 2 | 1 | 2 | 0.5 | 0.25 to 4 | 8 | 0.5-2* |
| Amoxicillin (16–0.25 mµ/ml) | >16 | >16 | >16 | >16 | 2 | 2 | 16 | 2 | 2 to >16 | 4 | N/A |
| Spectinomycin (64–8 mµ/ml) | 32 | <8 | 16 | <8 | 32 | 16 | 32 | <8 | <8 to 32 | >64 | 8-64* |
| Sulphadimethoxine (256–32 mµ/ml) | >256 | 128 | 256 | <128 | 256 | 256 | >256 | 128 | 128 to >256 | >256 | N/A |
| Trimethoprim/sulfamethoxazole (2/38–0.5/9.5 mµ/ml) | >2/38 | >2/38 | >2/38 | >2/38 | >2/38 | >2/38 | >2/38 | >2/38 | >2/38 | <0.5/9.5 | 0.03/0.6 – 0.25/4.8 |
| Florfenicol (8–0.25 mµ/ml) | 4 | <1 | 4 | <1 | 2 | <1 | 2 | <1 | <1 to 4 | 8 | 4-16 |
| Sulphathiazole (256–32 mµ/ml) | >256 | 64 | 256 | 64 | >256 | 64 | 256 | 64 | 64 to >256 | >256 | N/A |
| Penicillin (8–0.06 mµ/ml) | >8 | >8 | >8 | 4 | >8 | 4 | >8 | 4 | 4 to >8 | >8 | N/A |
| Streptomycin (1024–8 mµ/ml) | <8 | <8 | <8 | <8 | <8 | <8 | <8 | <8 | <8 | 16 | N/A |
| Novobiocin (4–0.5 mµ/ml) | 2 | 1 | 2 | <0.5 | 2 | 1 | 1 | <0.5 | <0.5–2 | >4 | N/A |
| Tylosin tartrate (20–2.5 mµ/ml) | >20 | 20 | >20 | 20 | >20 | 20 | >20 | 20 | 20 to >20 | >20 | N/A |
| Clyndamycin (4–0.5 mµ/ml) | >4 | >4 | >4 | >4 | >4 | >4 | >4 | >4 | >4 | >4 | N/A |
| Amikacin (64–8 mµ/ml) | <8 | <8 | <8 | <8 | <8 | <8 | <8 | <8 | <8 | 16 | 0.5-4* |
| Ampicillin (32–4 mµ/ml) | >32 | <4 | >32 | <4 | >32 | <4 | 16 | <4 | <4 to >32 | <4 | 2-16 |
| Ampicillin/sulbactam 2:1 ratio (4/2–32/16 mµ/ml) | 32/16 | < 4/2 | >32/16 | < 4/2 | >32/16 | < 4/2 | >32/16 | < 4/2 | <4/2 to >32/16 | <4/2 | N/A |
| Aztreonam (32–8 mµ/ml) | >32 | 32 | >32 | 16 | >32 | 32 | >32 | 16 | 16 to >32 | <8 | N/A |
| Cefazolin (32–4 mµ/ml) | >32 | 32 | >32 | 32 | >32 | 32 | >32 | 32 | 32 to >32 | <4 | 1-4* |
| Cefepime (32–4 mµ/ml) | >32 | <4 | >32 | <4 | >32 | <4 | >32 | <4 | <4 to >32 | <4 | N/A |
| Cefotetan Na (32–8 mµ/ml) | >32 | >32 | >32 | >32 | >32 | >32 | >32 | >32 | >32 | <8 | N/A |
| Ceftriaxone (64–1 mµ/ml) | >64 | <1 | >64 | <1 | >64 | 4 | >64 | <1 | <1 to >64 | <1 | N/A |
| Ceftazidime (32–1 mµ/ml) | >32 | <1 | >32 | <1 | >32 | <1 | >32 | <1 | <1 to >32 | <1 | N/A |
| Cefuroxime (32–4 mµ/ml) | >32 | 32 | >32 | 32 | >32 | 32 | >32 | 32 | 32 to >32 | 8 | N/A |
| Ciprofloxacin (4–0.5 mµ/ml) | <0.5 | <0.5 | <0.5 | <0.5 | <0.5 | <0.5 | <0.5 | <0.5 | <0.5 | <0.5 | N/A |
| Gatifloxacin (8–1 mµ/ml) | <1 | <1 | <1 | <1 | <1 | <1 | <1 | <1 | <1 | <1 | N/A |
| Meropenem (8–1 mµ/ml) | >8 | 4 | >8 | 2 | 8 | 4 | >8 | 2 | 2 to >8 | <1 | N/A |
| Imipenem (16–2 mµ/ml) | >16 | 8 | >16 | 16 | >16 | 16 | >16 | <2 | <2 to >16 | <2 | 0.06-0.25* |
| Nitrofurantoin (128–16 mµ/ml) | <16 | <16 | <16 | <16 | <16 | <16 | <16 | <16 | <16 | <16 | 4-16* |
| Cefoxitin (32–4 mµ/ml) | >32 | >32 | >32 | >32 | >32 | >32 | >32 | >32 | >32 | 8 | 2-8 |
| Piperacillin (128–16 mµ/ml) | >128 | <16 | >128 | <16 | >128 | >128 | >128 | <16 | <16 to >128 | <16 | N/A |
| Piperacillin/tazobactam constant 4 (128/4–16/4 mµ/ml) | >128/4 | <16/4 | >128/4 | <16/4 | >128/4 | <16/4 | >128/4 | <16/4 | <16/4 to >128/4 | <16/4 | N/A |
| Ticarcillin/clavulanic acid constant 2 (64/2–16/2 mµ/ml) | >64/2 | <16/2 | >64/2 | <16/2 | >64/2 | <16/2 | >64/2 | <16/2 | <16/2 to >64/2 | <16/2 | 4/2-16/2* |
| Tobramycin (8–4 mµ/ml) | <4 | <4 | <4 | <4 | <4 | <4 | <4 | <4 | <4 | <4 | N/A |
| Cefpodoxime (16–2 mµ/ml) | >16 | <2 | >16 | <2 | >16 | <2 | >16 | <2 | <2 to >16 | <2 | 0.25-1* |

*MIC values provided by the CLSI for when the test is run at 35 +/- 2 °C

| **Supplementary Table 3** Antimicrobial susceptibility test by the disc-diffusion (Kirby–Bauer) method for STIR-GUS-F2f7 and other *Fno* | | | | |
| --- | --- | --- | --- | --- |
| isolates in CHAH after 72h incubation, the mean zone diameters (mm) and standard deviation are presented | | | | |
| Antimicrobial (concentration) | STIR-GUS-F2f7 | Ehime-1 | STIR-MATT-F1f6 | PQ1104 |
| Enrofloxacin (5µg/disc) | 42 ± 3.46 | 40.5 ± 1 | 42.5 ± 1.91 | 43.5 ± 3.42 |
| Kanamycin (30µg/disc) | 35.3 ± 4.16 | 34.5 ± 4.12 | 38 ± 1.63 | 42.5 ± 1.91 |
| Gentamicin (2µg/disc) | 32 ± 2 | 29 ± 3.83 | 32.5 ± 1.91 | 33 ± 5.03 |
| Tetracycline (30µg/disc) | 22 ± 2 | 20 ± 2 | 23 ± 1.41 | 20 ± 2 |
| Oxytetracycline (30µg/disc) | 29.5 ± 4.43 | 26 ± 4.9 | 28.5 ± 1.91 | 27.5 ± 1.91 |
| Florfenicol (30µg/disc) | 22.8 ± 1.1 | 25.3 ± 1.15 | 24.8 ± 5.4 | 24.8 ± 2.28 |
| Oxolinic acid (2µg/disc) | 20.5 ± 4.73 | 18.5 ± 3.79 | 18.5 ± 4.12 | 21.5 ± 1.91 |
| Streptomycin (10µg/disc) | 27.5 ± 1.91 | 23 ± 1.41 | 22 ± 2 | 23 ± 1.41 |
| Penicillin G (10units ) | 0 | 0 | 0 | 0 |
| Lincomycin (15µg/disc) | 0 | 0 | 0 | 0 |
| Oleandomycin (15µg/disc) | 0 | 0 | 0 | 0 |
| Amoxicillin (10µg/disc) | 0 | 0 | 0 | 0 |
| Carbenicillin (100µg/disc) | 0 | 0 | 0 | 0 |
| Polymyxin B (300 units) | 0 | 0 | 0 | 0 |
| Ampicillin (10 µg/disc) | 0 | 0 | 0 | 0 |
| Sulphamethoxazole/ trimethoprim 19:1 (25µg/disc) | 0 | 0 | 0 | 0 |

| **Supplementary Table 4** Sequence similarity in percentage (%) between STIR-GUS-F2f7 and other members of the genus | | | | | | |
| --- | --- | --- | --- | --- | --- | --- |
| Gene | *Fno* | *Fnn* | *F. philomragia* | *F. tularensis** | *F. halioticida* |  |
| *dnaA* | 99-100 | 99 | 94-95 | 85-86 | 84 |  |
| *mutS* | 100 | 93-94 | 92-93 | 76-77 | 75 |  |
| *prfB* | 99-100 | 94 | 93-95 | 84-86 | 81 |  |
| *putA* | 99 | 96 | 96-97 | 80-81 | 75 |  |
| *rpoA* | 99 | 99 | 92-93 | 83-84 | 83 |  |
| *rpoB* | 99 | 91 | 92 | 86 | 84 |  |
| *tpiA* | 99-100 | 98 | 94-96 | 78-80 | 79 |  |
| *mdh* | 100 | 98-99 | 96 | 84-86 | 85 |  |
| 16SrRNA+ITS+23SrRNA | 99-100 | 98-99 | 99 | 97 | 98 |  |
| * Includes data from isolates of the four *F. tularensis* subspecies | | | | | | |

| **Supplementary Table 5** Evolutionary models used on the phylogenetic analyses | | | | |
| --- | --- | --- | --- | --- |
| Gene-sequence | Model | Rates among sites | positions | sequences |
| 16SrRNA | K2 | Discrete gamma distribution | 1350 | 50 |
| dnaA | T92 | Discrete gamma distribution | 1298 | 12 |
| mdh | T92 | Uniform rates | 576 | 12 |
| mutS | TN93 | Discrete gamma distribution | 2326 | 13 |
| prfB | HKY | Discrete gamma distribution | 880 | 12 |
| putA | HKY | Discrete gamma distribution | 2333 | 12 |
| rpoA | T92 | Discrete gamma distribution | 464 | 17 |
| rpoB | TN93 | Discrete gamma distribution | 3042 | 13 |
| tpiA | T92 | Discrete gamma distribution | 507 | 15 |
| 16SrRNA+ITS+23SrRNA | HKY | Uniform rates | 2676 | 9 |
| Concatenated sequence | GTR | Discrete gamma distribution | 15750 | 22 |
